# Mangrove-Associated Fungi: A Novel Source of Potential Anticancer Compounds

**DOI:** 10.3390/jof4030101

**Published:** 2018-08-24

**Authors:** Sunil K. Deshmukh, Manish K. Gupta, Ved Prakash, M. Sudhakara Reddy

**Affiliations:** 1 TERI-Deakin Nano Biotechnology Centre, The Energy and Resources Institute (TERI), Darbari Seth Block, IHC Complex, Lodhi Road, New Delhi 110003, India; manish.gupta@teri.res.in; 2 Department of Biotechnology, Motilal Nehru National Institute of Technology, Allahabad 211004, India; ved.mits@gmail.com; 3 Department of Biotechnology, Thapar Institute of Engineering & Technology, Patiala, Punjab 147004, India; msreddy@thapar.edu

**Keywords:** endophytic fungi, anticancer compounds, mangroves, co-culture, epigenetic modification

## Abstract

Cancer is the second leading cause of death worldwide, and the number of cases is increasing alarmingly every year. Current research focuses on the development of novel chemotherapeutic drugs derived from natural as well as synthetic sources. The abundance and diversity in natural resources offer tremendous potential for the discovery of novel molecules with unique mechanisms for cancer therapy. Mangrove-derived fungi are rich source of novel metabolites, comprising novel structure classes with diverse biological activities. Across the globe, coastal areas are primarily dominated by mangrove forests, which offer an intensely complex environment and species that mostly remain unexplored. In recent years, many structurally diverse compounds with unique skeletons have been identified from mangrove fungi and evaluated for their antiproliferative properties. These compounds may serve as lead molecules for the development of new anticancer drugs. Mangrove endophytes can be modulated using epigenetic means or culture optimization methods to improve the yield or to produce various similar analogs. The present review provides an insight into the bioactive metabolites from mangrove endophytes reported during the period from 2012 to 2018 (up to April, 2018) along with their cytotoxic properties, focusing on their chemical structures and mode of action, as indicated in the literature.

## 1. Introduction

Mangroves are salt-tolerant forest ecosystems, representing a lively ecosystem with an amalgam of land-dwelling and marine habitats with high biodiversity and socio-economic importance [1]. Marine fungi are among the most prominent species existing in mangrove forests, and support nutrient replenishment [2]. As per reports on marine fungi, in this ecological niche mangrove fungi make up the second largest group [3]. These fungi may occur as saprophytes, symbiotically or as a parasites in the mangrove ecosystem. In addition, these fungi belong to both the lower class, such as oomycetes and thraustochytrids, and the upper class, such as ascomycetes and basidiomycetes. Fungal secondary metabolites are structurally quite diverse, and their functions mostly depend on self-defense against other microorganisms [4]. Most often mangrove fungi flourish in challenging habitats, making them a rich source of bioactive metabolites. Endophytes are one of the various groups of mangrove fungi that have resulted in the identification of a large number of new bioactive metabolites of nutraceutical and pharmaceutical importance. These include antibiotic, anticancer, antidiabetic, antioxidant, antiviral, anti-inflammatory and immunosuppressive drugs, along with other pharmaceutical agents [5].

Cancer affects different organs, and is identified by the unchecked proliferation of abnormal cells that invade other healthy tissue. The treatment is primarily confined to chemotherapy. Besides being expensive, chemotherapy is known to cause severe side effects, making treatment problematic. The non-effectiveness of many existing drugs along with multi-drug resistance further aggravates the problem, making cancer treatment difficult. For medicinal chemists, the primary goal remains the discovery and identification of chemotherapeutic agents derived from natural products. Secondary metabolites have opened new avenues for the development of novel therapeutic agents [6,7]. Endophytic fungi, which are a less-explored area of the microbial community, have a tremendous potential to produce new metabolites that can be used for pharmaceutical applications. Since the initial report of the identification of paclitaxel, derived from an endophyte associated with Northwest Pacific yew by Stierle et al. [8], scientists have identified many other crucial anticancer molecules from fungal endophytes [6]. Many researchers were attracted to marine mangrove fungi because of their diversity, which may lead to the discovery of several novel natural products. With the remarkable advancements in spectroscopic techniques, separation methods and microplate-based sensitive in vitro assays, the natural product exploration of mangrove fungi has attracted special attention regarding novel and unexplored chemical scaffolds [9]. Of the various existing groups of mangrove fungi, endophytes have been identified as producers of new bioactive metabolites with pharmaceutical and nutraceutical importance.

Most of the endophytes have the potential to produce novel bioactive metabolites, which will undoubtedly boost novel drug discovery. However, higher similarity among microbes leads to the frequent identification of the same compound in the endophytes. During axenic cultivation, a specific portion of the biosynthetic genes are expressed while growing in vitro, and various genes stay masked or silent and do not express in laboratory conditions. For this reason, the routine method of fermentation yields metabolites without chemical diversity. Co-cultivation could help to overcome this problem and is preferred, with two or more microbes allowed to grow together. This approach offers a better competitive environment, allowing the increased production of constitutive as well as cryptic compounds that are not traced out in axenic cultures [10]. Several co-cultivation strategies such as different combinations of fungi, the co-cultivation of fungi with bacteria and the co-cultivation of different bacteria have been reported for the enhancement of the chemical diversity of marine-derived microorganisms [10].

The development of methodologies to induce the expression of biosynthetic transcription as well as the suppression of these genes plays a vital role in the search for new secondary metabolites. The regulation of the enzymes that control metabolite production can be achieved by changing epigenetic mechanisms such as DNA methylation and histone modifications (acetylation and phosphorylation) by using epigenetic modifiers [11]. As an example of the importance of epigenetic modulation in producing unknown natural products, when *Aspergillus niger* is cultivated over a two-week period in vermiculite-based semi-solid medium treated with suberoylanilide hydoxamic acid (SAHA), it leads to the isolation of a new fungal metabolite nygerone [12]. Hence the epigenetic approach can be a game changer in the production/enhancement of secondary metabolites.

**Figure 1:**
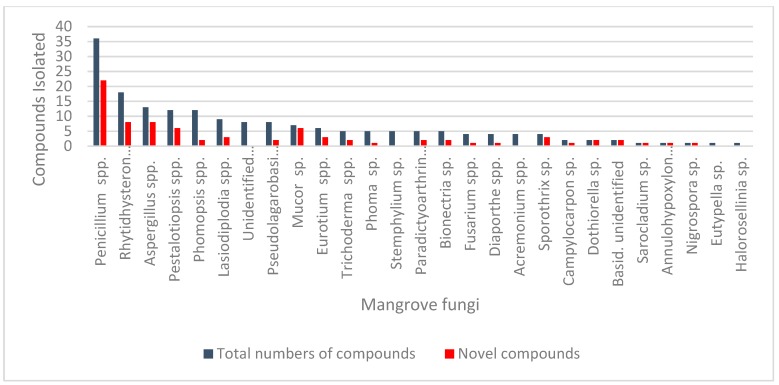
Novel anticancer bioactive compounds reported from mangrove fungi.

The present review provides a comprehensive overview of the bioactive metabolites identified from mangrove endophytes during the period from 2012 to 2018 (up to April, 2018) including eighty novel compounds of the total 181 reported. The total number of compounds as well as novel compounds isolated from mangrove fungi during this period is presented in Figure 1. The origin, chemical structure of the biological targets and efficacies of these compounds are also discussed where available. The anticancer properties of many of these compounds are presented in Table 1. They are arranged based on the broader category of the taxonomic class of the cytotoxic compounds producing fungi. An attempt has also been made to review recent developments such as co-cultivation and epigenetic modifications in endophytic fungi to enhance the secondary metabolite production.

## 2. Bioactive Compounds in Mangrove Plants

### 2.1. Compounds Produced by Coelomycetes

**Figure 2:**
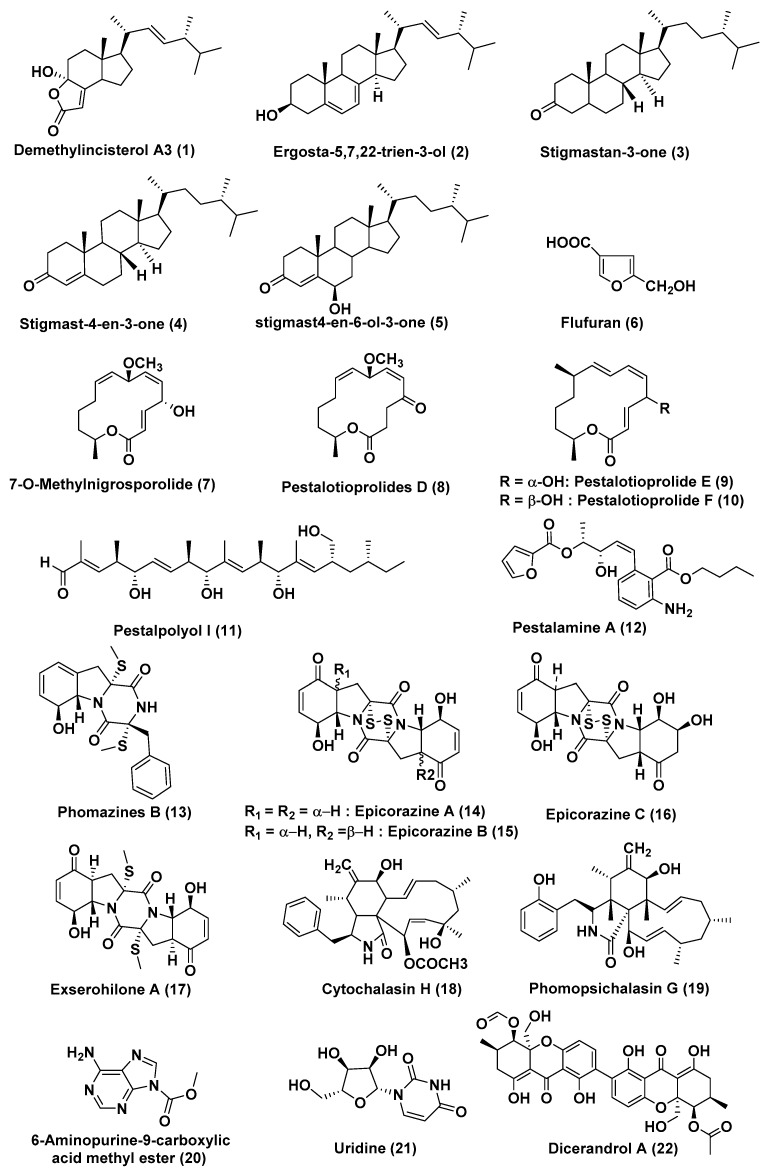
Structures of metabolites isolated from Coelomycetes (**1**–**22**).

*Pestalotiopsis* is the most noteworthy coelomycetous fungi and the species of *Pestalotiopsis* are known to produce the diverse array of novel compounds. Strobel and Long [13] described *Pestalotiopsis* as the “*E. coli* of the temperate and tropical rainforest systems”. The species *Pestalotiopsis* is widely recognized to be a prolific producer of a diverse array of metabolites that include alkaloids, chromones, coumarins, isocoumarin derivatives, lactones, peptides, phenols, phenolic acids, quinones, semiquinones, xanthones, terpenoids and xanthone derivatives along with an array of antimicrobial, antifungal, antitumor, antiviral, antineoplastic, and antioxidant compounds [14,15]. Some of the cytotoxic compounds reported from this genus such as demethylincisterol A3 (**1**), ergosta-5,7,22-trien-3-ol (**2**), stigmastan-3-one (**3**), stigmast-4-en-3-one (**4**), stigmast4-en-6-ol-3-one (**5**), and flufuran (**6**) (Figure 2), were discovered from *Pestalotiopsis* spp., associated with Chinese mangrove *Rhizophora mucronata*. Compounds **2**–**6** showed cytotoxicity against human cancer cell lines HeLa, A549, and HepG, with IC_50_ values in the range of 11.44–102.11 µM. Compound **1** had the most potential, with IC_50_ values reaching the nM activity level from 0.17 to 14.16 nM. Flow cytometric investigation demonstrated that compound 1 inhibited the cell cycle at the G0/G1 phase in a dose-dependent manner with a significant induction of apoptosis on the three tested cell lines. The involvement of the mitochondria in compound-**1**-induced apoptosis was demonstrated using MMP [16].

The compounds 7-*O*-methylnigrosporolide (**7**), pestalotioprolides D–F (**8**, **9**, **10**) (Figure 2), were extracted from the *Pestalotiopsis microspora*, endophytic fungus obtained from the fruits of *Drepanocarpus lunatus* collected from Douala, Cameroon. An approximately ten-fold increase in the yield of compounds **9** and **10** compared to axenic fungal control was observed when *P. microspora* was co-cultured with *Streptomyces lividans*. Compounds **7**–**10** exhibited cytotoxicity against the L5178Y cell line with IC_50_ values of 0.7, 5.6, 3.4, and 3.9 µM, respectively, and compound **9** also showed potent activity against the A2780 cell line displaying an IC_50_ value of 1.2 µM [17].

Another study by Hemphill et al. reported a new compound pestalpolyol I (**11**) (Figure 2) of the polyketide group from *Pestalotiopsis clavispora*, the endophytic fungus obtained from petioles of the *Rhizophora harrisonii*, growing in Port Harcourt (Nigeria). Compound **11** showed cytotoxicity against the L5178Y cell line with an IC_50_ value of 4.10 µM [18].

A new aromatic amine, pestalamine A (**12**) (Figure 2), was isolated from *P. vaccinia* from a branch of *Kandelia candel*, a viviparous mangrove species widely distributed in coastal and estuarine areas of southern China. The structure of pestalamine A **12** was determined by spectroscopic methods, especially 2D NMR analyses. Compound **12** showed moderate cytotoxicity against MCF-7, HeLa, and HepG2 human cancer cell lines with IC_50_ values of 40.3, 22.0, and 32.8 µM, respectively [19].

Phomazines B (**13**), epicorazine A (**14**), epicorazine B (**15**), epicorazine C (**16**), exserohilone A (**17**) (Figure 2), were isolated from an endophytic fungus, *Phoma* sp. OUCMDZ-1847 associated with the fruit of *Kandelia candel* collected in Wenchang, Hainan Province, China. Compounds **13**–**17** showed cytotoxicity against the HL-60, HCT-116, K562, MGC-803, and A549 cell lines with IC_50_ values in the range of 0.05 to 8.5 µM [20].

Cytochalasin H (**18**) (Figure 2), was identified form *Phomopsis* sp., an endophytic fungus of mangrove origin in Zhanjiang, China. Compound **18** was shown to inhibit the cell cycle of A549 cells at the G2/M phase. Additionally, DNA fragmentation along with a decrease in the transmembrane potential of mitochondria was observed in A549 cells. It was also shown to regulate the expression level of Bax, P53, Bcl-xL, and Bcl-2. On treatment with Cytochalasin H, the migration capability was impaired in a dose-dependent manner [21].

A new cytochalasin, phomopsichalasin G (**19**) (Figure 2), was isolated from *Phomopsis* sp. xy21, and xy22, associated with *Xylocarpus granatum*, collected in Trang Province, Thailand. Compound **19** exhibited inhibitory activities against HCT-8, HCT-8/T, A549, MDA-MB-231, and A2780 cancer cell lines with IC_50_ values of 7.5, 8.6, 6.4, 3.4, and 7.1 μM, respectively [22].

6-Aminopurine-9-carboxylic acid methyl ester (**20**) and uridine (**21**) (Figure 2), were isolated from *Phomopsis longicolla* HL-2232, an endophyte of *Bruguiera sexangula* var. *rhynchopetala*. The compounds **20** and **21** exhibited cytotoxicity against MCF-7 and A549 cell lines with IC_50_ values of 14.9 and 8.6 μM, respectively [23].

**Figure 3:**
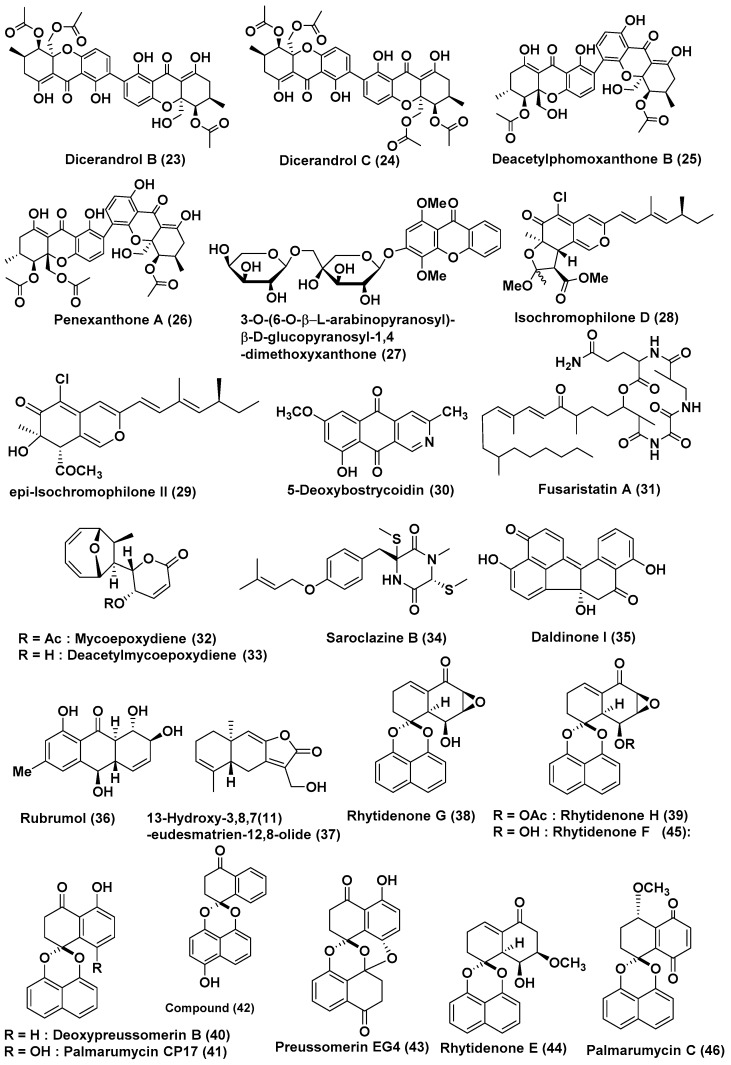
Structures of metabolites isolated from Coelomycetes (**23**–**33**) and Ascomycetes (**34**–**46**).

*Phomopsis* sp. HNY29-2B, an endophyte isolated from the branch of *Acanthus llicifolius* collected from the South China Sea in Hainan province, China, is reported as a source of the known phomoxanthones, including dicerandrol A (**22**) (Figure 2), dicerandrol B (**23**), dicerandrol C (**24**), diacetylphomoxanthone B (**25**) and penexanthone A (**26**) (Figure 3). Compound **22** exhibited broad-spectrum cytotoxic activity against MDA-MB-435, HCT-116, Calu-3 and Huh7 cell lines with IC_50_ values of 3.03, 2.64, 1.76 and 4.19 μM, respectively. Compound **23** and **26** showed potent cytotoxic activities against MDA-MB-435, HCT-116, and Calu-3 (IC_50_ < 10 μM), and displayed poor cytotoxic activity effects against the MCF-10A cell line (IC_50_ > 50 μM). Compound **24** exhibited cytotoxic activity against the MDA-MB-435, HCT-116, Calu-3, MCF-10A cell lines with IC_50_ values of 44.10, 42.63, 36.52, and 33.05 μM, respectively. Compound **25** exhibited cytotoxicity against the MDA-MB-435, HCT-116, Calu-3, Huh7 cell lines with IC_50_ values of 14.40, 7.12, 4.14 and 29.20 μM and showed no cytotoxic effect on the MCF-10A cell line [24].

A new xanthone *O*-glycoside, 3-*O*-(6-*O*-α-l-arabinopyranosyl)-β-d–glucopyranosyl-1,4-dimethoxyxanthone (**27**) (Figure 3), was isolated from an endophytic fungus, *Phomopsis* sp. (ZH76) obtained from the stem of *Excoecaria agallocha* of a mangrove from Dong Sai on the South China Sea coast. Compound **27** had an inhibitory effect on the growth of HEp-2 and HepG2 cells displaying IC_50_ values of 9 and 16 μM, respectively [25].

A highly oxygenated chloroazaphilone derivative, isochromophilone D (**28**), and a known analogue epi-Isochromophilone II (**29**) (Figure 3), were isolated from *Diaporthe* sp. SCSIO 41011, the endophytic fungus associated with *Rhizophora stylosa*, collected in Sanya city, Hainan Province, China. Compound **29** exhibited cytotoxic activity against the ACHN, OS-RC-2, and 786-O cell lines with IC_50_ values ranging from 3.0 to 4.4 μM. Sorafenib, a positive control, exhibited cytotoxic activity against the tested cell lines with IC_50_ in the range of 3.4 to 7.0 μM. Compound **28** showed activity against 786-O cells with an IC_50_ of 8.9 μM. In a dose- and time-dependent manner, apoptosis was induced in 786-O cells by compound **28** [26].

New chromeno[3,2-c] pyridine, 5-deoxybostrycoidin (**30**), and fusaristatin A (**31**) (Figure 3), were isolated from an endophytic fungus *Diaporthe phaseolorum* SKS019 isolated from the branches of the mangrove plant *Acanthus ilicifolius*, collected from Shankou in Guangxi province, China. Compound **30** exhibited cytotoxicity against the MDA-MB-435, and NCI-H460 human cancer cell lines with IC_50_ values of 5.32 and 6.57 μM, respectively, and compound **31** showed growth-inhibitory activity against the MDA-MB-435 human cancer cell line with an IC_50_ value of 8.15 μM [27].

Mycoepoxydiene (**32**) and deacetylmycoepoxydiene (**33**) (Figure 3) were isolated from a mangrove endophytic fungus *Phomosis* sp. A818 isolated from the foliage of *Kandelia candel*, collected from the Mangrove Nature Conservation Area of Fugong, Fujian Province, China. Compounds **32** and **33** had IC_50_ values of 7.85 and 14.61 µM, respectively, against MDA-MB-435 [28]. Mycoepoxydiene (**32**) (Figure 3), significantly suppressed antigen-stimulated degranulation and cytokine production in mast cells and IgE-mediated passive cutaneous anaphylaxis in mice. Compound **32** suppressed antigen-induced activation of Syk, and subsequently inhibited the phosphorylation of PLCγ1, Akt, and MAPKs such as extracellular signal-regulated kinase, c-jun N-terminal kinase, and p38 in mast cells. Mycoepoxydiene can inhibit the activation of mast cells and protect mice from a mast-cell-mediated allergic response through inhibiting the activation of Syk [29].

### 2.2. Compounds Produced by Ascomycetes

A new diketopiperazine derivative, saroclazine B (**34**) (Figure 3), was isolated from the mangrove-derived fungus *Sarocladium kiliense* HDN11-84 isolated from the rhizosphere soil of the mangrove plant *Thespesia populnea*, collected in Guangxi Province, China. Compound **34** showed cytotoxicity against HeLa cell lines with an IC_50_ value of 4.2 μM [30].

Benzofluoranthene metabolites and daldinone I (**35**) (Figure 3) were extracted from *Annulohypoxylon* sp., an endophytic fungus associated with *Rhizophora racemose*, collected in Cameroon. Compound **35** exhibited average to potent cytotoxicity with IC_50_ values of 14.1 and 6.6 µM, against Jurkat J16 and Ramos cell lines, respectively. It was reported that compound **35** induces apoptotic cell death caused by the induction of intrinsic apoptosis [31].

A new anthraquinone rubrumol (**36**) (Figure 3) with poly-hydroxyl groups was isolated from a halo-tolerant endophytic fungus *Eurotium rubrum*, isolated from the salt-tolerant wild plant *Suaeda salsa* L. collected from the “BoHai” seaside, China. The biological effect of compound **36** on Topo I to relax supercoiled pBR322 DNA was investigated in the cleavable complex assay. The results indicated that compound **36** displayed biological activity compared to the positive control camptothecin. The relaxation activity of rubrumol (**36**) was stronger than that of camptothecin at the concentration of 100 μM. The band backward shifting and trailing of rubrumol (**36**) was observed at 100, 50, 10, 5 and 1 μM. Compound **36** also exhibited cytotoxic activities against A549, MDA-MB-231, PANC-1 and HepG2 human cancer cell lines, by MTT method. The inhibition rate for compound **36** against these four cancer cell line was less than 60% at 100 µg/mL, which implied that it displayed no significant cytotoxic activity [32].

The 13-Hydroxy-3,8,7(11)-eudesmatrien-12, 8-olide (**37**) (Figure 3) was isolated from *Eutypella* sp. 1–15 isolated from the soil of the mangrove rhizosphere in Jimei, Fujian Province, China. Compound **37** exhibited potent anticancer activity against JEKO-1 and HepG2 with IC_50_ values of 8.4 and 28.5 Μm, respectively [33].

**Figure 4:**
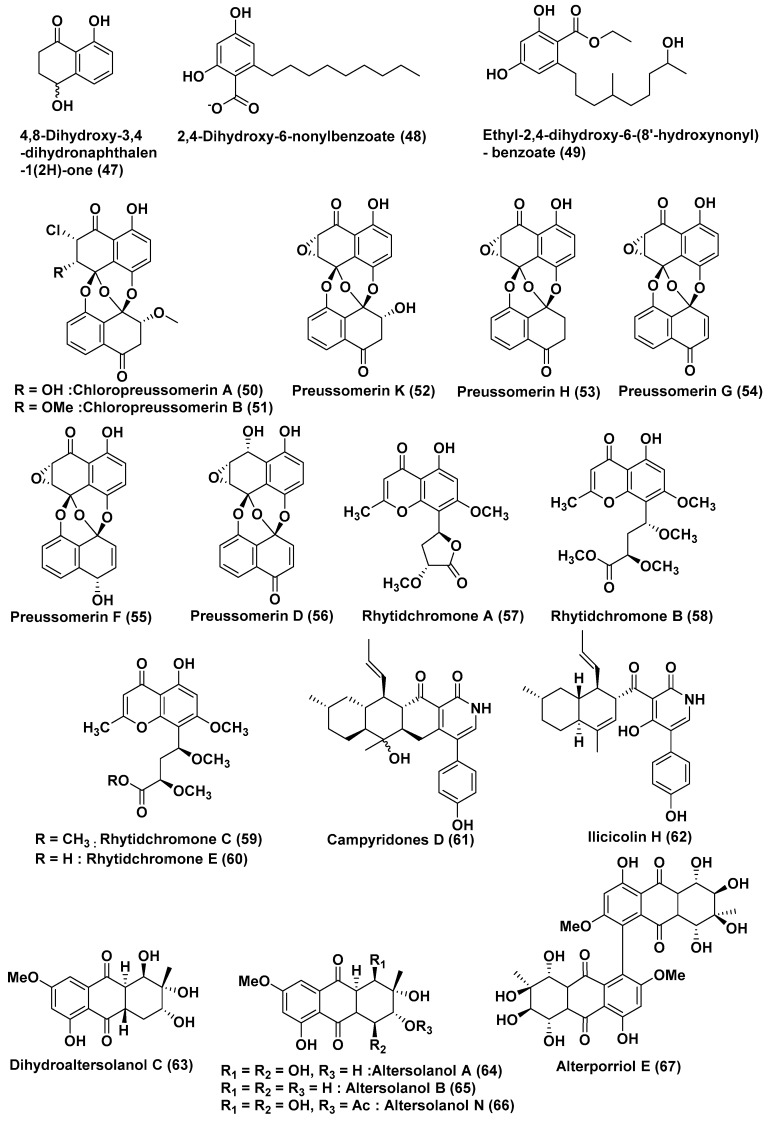
Structures of metabolites isolated from Ascomycetes (**47**–**67**).

Rhytidenones G (**38**), H (**39**), deoxypreussomerin B (**40**), palmarumycin CP17 (**41**), 1-oxo-1,4-dihydronapthalene-4-spiro-20-naptho[400-hydroxy-100,800-de][10,30]-dioxine (**42**), preussomerin EG4 (**43**), rhytidenone E (**44**), rhytidenone F (**45**), palmarumycin C5 (**46**), (Figure 3), and 4,8-dihydroxy-3,4-dihydronaphthalen-1(2*H*)-one (**47**) (Figure 4), were isolated from *Rhytidhysteron rufulum* AS21B, an endophytic fungus associated with the leaves of *Azima sarmentosa*, collected from the mangrove forest in Samutsakhon province, Thailand. The culture in acidic medium enhanced the production of compounds **41** and **45**, with a four-fold and eight-fold increase, respectively, which are present in minor quantities under normal culture condition. Compounds **38**–**47** exhibited cytotoxicity against Ramos cells with IC_50_ values of 17.98, 0.018, 18.00, 33.1, 15, 82.9, 0.461, 0.048, 31.7 and 23.1 µM, respectively, while the control Ibrutinib exhibited cytotocity of 28.7 µM against the same cell line. Compounds **38**–**39**, **44**–**45**, and **47** exhibited cytotoxicity against the H1975 cell lines with an IC_50_ value of 7.3, 0.252, 10.24, 1.17 and 50 µM, respectively, while control afatinib exhibited cytotoxicity of 1.97 µM against the same cell line [34].

*Lasiodiplodia* sp. 318#, an endophytic fungus associated with *Excoecaria agallocha*, collected from Guangdong Province, China, was the source of compound 2,4-Dihydroxy-6-nonylbenzoate (**48**) (Figure 4). Compound **48** exhibited cytotoxicity against the MMQ and GH3 cell lines with IC_50_ values of 5.2 and 13.0 μM, respectively [35]. Previously, a new lasiodiplodin—ethyl-2,4-dihydroxy-6-(8’-hydroxynonyl)-benzoate (**49**)—was obtained from the same fungus. Compound **49** exhibited average cytotoxicity against the MDA-MB-435, HepG2, HCT-116, A549, and leukaemia THP1 cell lines with IC_50_ values of 10.13, 12.50, 11.92, 13.31 and 39.74 µM, respectively [36].

Two new chlorinated preussomerins, chloropreussomerins A (**50**), and B (**51**), and a known preussomerin analog preussomerin K (**52**), preussomerin H (**53**), preussomerin G (**54**), preussomerin F (**55**), preussomerin D (**56**) (Figure 4), were obtained from the endophytic fungus *Lasiodiplodia theobromae* ZJ-HQ1 isolated from *Excoecaria agallocha* collected from Guangdong Province, China. Compounds **50**–**51** and **56** were found to be active against the A549 and MCF-7 cell lines with IC_50_ values ranging from 5.9–8.9 μM, and compounds **52**–**55** showed cytotoxicity against A549, HepG2, and MCF-7 human cancer cell lines with IC_50_ values of 2.5–9.4 µM [37].

Four highly oxygenated chromones—rhytidchromone A (**57**), B (**58**), C (**59**) and E (**60**) (Figure 4)—were obtained from *Rhytidhysteron rufulum* BG2-Y, an endophyte associated with the leaves of *Bruguiera gymnorrhiza* collected from Prachuab Kiri Khan Province, Thailand. Compounds **57**–**60** were found to be active against Kato-3 cell lines, with IC_50_ values in the range of 16.0–23.3 µM, while the rhytidchromones A and C were active, with IC_50_ values of 19.3 and 17.7 µM respectively, against MCF-7 cell lines [38].

Campyridone D (**61**) and ilicicolin H (**62**) (Figure 4) were extracted from *Campylocarpon* sp. HDN13-307, an endophyte associated with the root of the mangrove plant *Sonneratia caseolaris*. Compounds **61** and **62** showed cytotoxicity, with the IC_50_ values of 8.8 µM and 4.7 µM, respectively, against HeLa cells [39].

*Stemphylium globuliferum*, an endophytic fungus associated with the Egyptian mangrove plant *Avicennia marina*, was the source of dihydroaltersolanol C (**63**), altersolanols A, B, N (**64**, **65**, **66**), and alterporriol E (**67**) (Figure 4) [40]. Compounds **63**, **64**, **65**, and **67** showed cytotoxicity with IC_50_ values of 3.4, 2.5, 3.7 and 6.9 µM, respectively, towards L5178Y cells [41]. Compound **66** also showed good activity, with IC_50_ values in the low micro-molar range towards L5178Y cells [42]. Mishra et al. [43] reported that compound **64** exhibited cytotoxicity against 34 human cancer cell lines in vitro, with mean IC_50_ (IC_70_) values of 0.005 µg/mL (0.024 µg/mL). It has also been reported that compound **64** is a kinase inhibitor and induces cell death by apoptosis through the caspase-dependent pathway, and that kinase inhibition might be the mechanism for the cytotoxic activity [44]. The pro-apoptotic and anti-invasive activity of compound **64** that occurred through the inhibition of the NF-κB transcriptional activity may be responsible for its antitumor potential [45].

**Figure 5:**
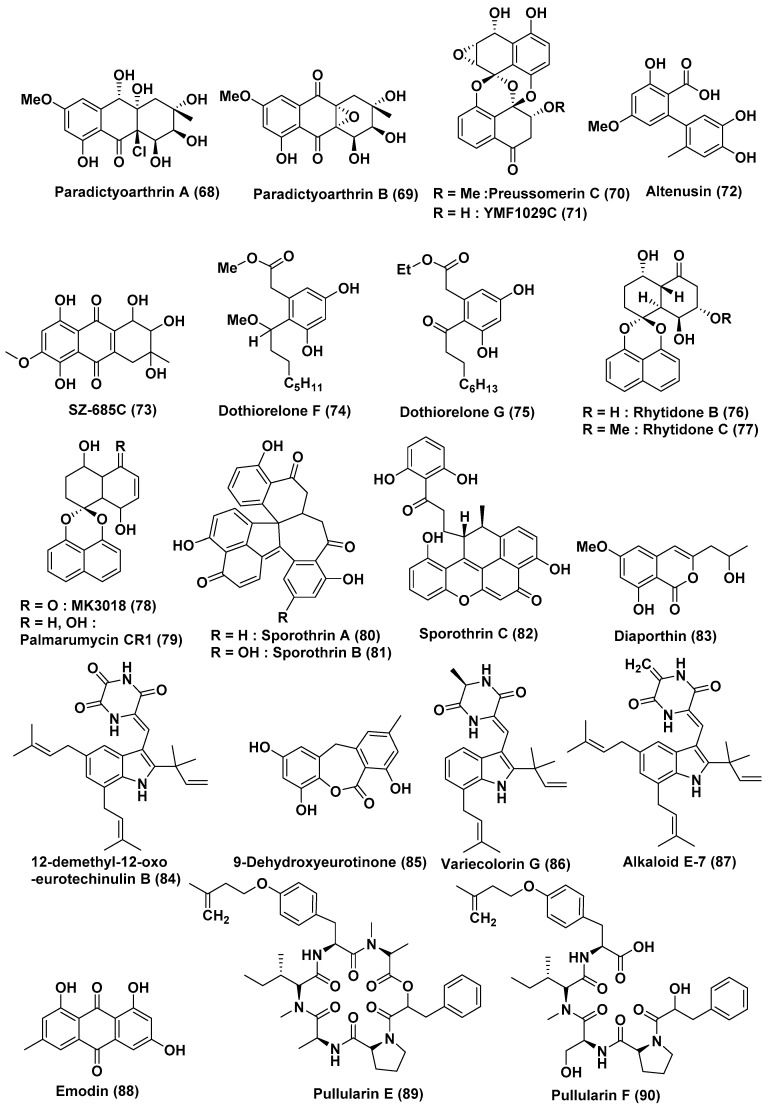
Structures of metabolites isolated from Ascomycetes (**68**–**90**).

Two new hydroanthraquinones, paradictyoarthrins A (**68**) and B (**69**) and the known compounds, preussomerin C (**70**), ymf 1029C (**71**) and altenusin (**72**) (Figure 5), were isolated from *Paradictyoarthrinium diffractum* BCC 8704, an endophyte associated with mangrove wood in Laem Son National Park, Ranong Province, Thailand. Compounds **68**–**72** were evaluated for cytotoxic activity against cancer cell-lines, KB, MCF-7, and NCI-H187, and noncancerous Vero cells. Compound **69** exhibited moderate cytotoxicity against KB, MCF-7 NCI-H187 and Vero cell lines with IC_50_ of 3.1, 3.8, 9.5 and 5.6 µg/mL, respectively, whereas Compound **68** showed weaker activity with IC_50_ values in the range of 23–31 µg/mL. Compounds **70**–**72** showed average to poor activity in tested cell lines. Compound ymf 1029C **71** showed relatively stronger cytotoxicity on NCI-H187 cells than other cell-lines (IC_50_ 5.0 µg/mL) [46].

A marine anthraquinone derivative SZ-685C (**73**) (Figure 5) has been isolated from the mangrove endophytic fungus *Halorosellinia* sp. (No. 1403), which was found in the South China Sea. The IC_50s_ of SZ-685C in nonfunctioning pituitary adenoma (NFPA), MMQ, and RPC cells were 18.76, 14.51, and 56.09 µM, respectively. Hoechst 33342 dye/propidium iodide (PI) double staining and fluorescein isothiocyanate-conjugated Annexin V/PI (Annexin V-FITC/PI) apoptosis assays detected an enhanced the rate of apoptosis in cells treated with SZ-685C. Enhanced expression levels of caspase 3 and phosphate and tensin homologs were determined by Western blotting. The protein expression levels of Akt were decreased when the primary human NFPA cells were treated with SZ-685C. It has been observed that SZ-685C (**73**) induces the apoptosis of human NFPA cells through the inhibition of the Akt pathway in vitro. These findings suggest that SZ-685C may be a potentially promising Akt inhibitor and anti-cancer agent for the treatment of NFPA [47].

SZ-685C (**73**), was previously reported to inhibit the proliferation of certain tumor cells. SZ-685C inhibited MMQ cell growth in a dose-dependent manner but showed little toxicity toward rat pituitary cells. The IC_50_ of SZ-685C in MMQ cells and RPCs were 13.2 and 49.1 µM, respectively. Increasing numbers of apoptotic cells were observed in response to escalating concentrations of SZ-685C, and the expression level of prolactin was inhibited. Nevertheless, the level of prolactin mRNA was unchanged. Additionally, miR-200c was upregulated in MMQ cells compared with RPCs, and downregulation of miR-200c was observed in SZ-685C-treated MMQ cells. Furthermore, the overexpression of miR-200c weakened the effect of the SZ-685C-induced apoptosis of MMQ cells. It has been suggested that SZ-685C induces MMQ cell apoptosis in a miR-200c-dependent manner [48].

Two new polyketides, named dothiorelons F (**74**) and G (**75**) (Figure 5), were isolated from *Dothiorella* sp., an endophytic fungus associated with the bark of the mangrove tree *Aegiceras corniculatum* at the estuary of Jiulong River, Fujian Province, China. Compounds **74** and **75** showed significant cytotoxicity against the Raji cancer cell line, with an IC_50_ value of 2 µg/mL [49].

New spironaphthalenes, rhytidones B–C (**76**, **77**) and known MK3018 (**78**), palmarumycin CR1 (**79**) (Figure 5), were extracted from *Rhytidhysteron* sp. an endophytic fungus associated with the leaves of *Azima sarmentosa*, collected from the mangrove forest in Samutsakhon province, Thailand. Compound **76** was found to be poorly active against CaSki cells, with an IC_50_ value of 22.81 μM, while compounds **77**–**79** showed average activity against the MCF-7 and CaSkicell lines with IC_50_ values in the range of 14.47 and 25.59 µM [50].

*Sporothrix* sp. isolated from the bark of an inshore mangrove sample *Kandelia candel* in the South China Sea was the source of Sporothrin A (**80**), sporothrin B (**81**), sporothrin C (**82**), diaporthin (**83**) (Figure 5). Compounds **81**–**83** were found to show weak cytotoxic activity with IC_50_ values of 20, 23, and 23 g/mL, respectively. Compound **80** exhibited strong inhibition of AChE in vitro (IC_50_ was 1.05 μM) [51].

A new dioxopiperazine alkaloid, 12-demethyl-12-oxo-eurotechinulin B (**84**), and one new anthraquinone derivative 9-dehydroxyeurotinone (**85**), and known compounds, variecolorin G (**86**), alkaloid E-7 (**87**), and emodin (**88**) (Figure 5), were isolated from *Eurotium rubrum*, an endophytic fungus associated with the inner tissue of *Hibiscus tiliaceus*, collected from Hainan Island, China. Compounds **84**–**88** displayed cytotoxic activity against one or two of the MCF-7, SW1990, HepG2, NCI-H460, SMMC7721, Hela, and Du145 cell lines in the range of 15–30 mg/mL [52].

**Figure 6:**
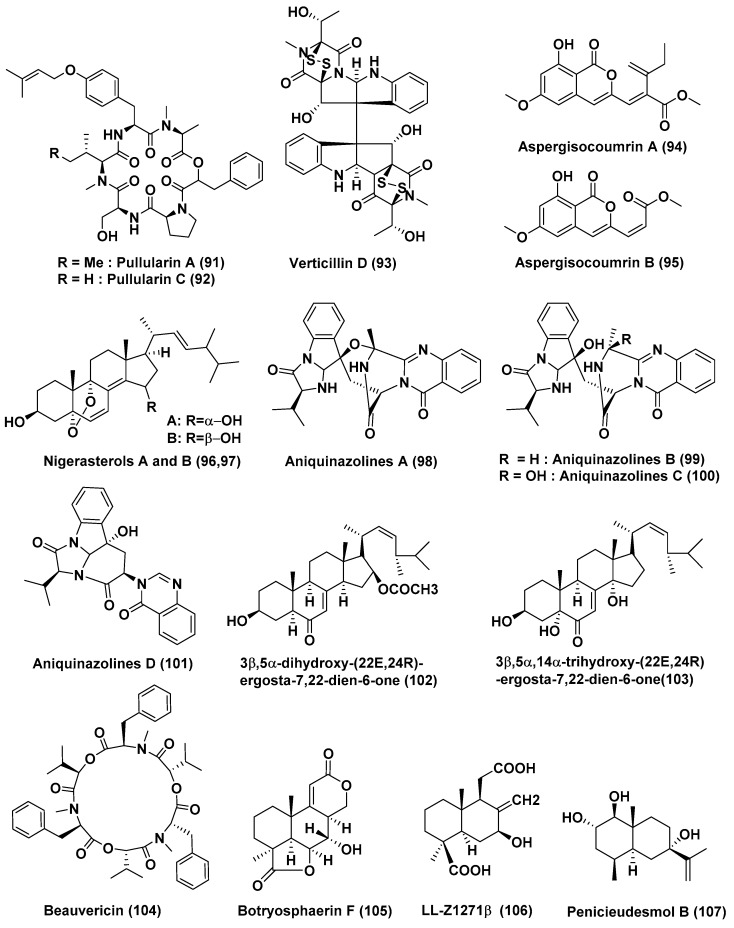
Structures of metabolites isolated from Ascomycetes (**91**–**93**) and Hyphomycetes (**94**–**107**).

Two new peptides—pullularins E (**89**) and F (**90**) (Figure 5)—and three known compounds—pullularins A (**91**) and C (**92**) and verticillin D (**93**) (Figure 6)—were extracted from *Bionectria ochroleuca*, an endophytic fungus associated with the inner leaf tissues of *Sonneratia caseolaris* from Hainan island, China. Compound **93** showed potent to moderate activity against L5178Y cell lines with an EC_50_ value of <0.1 µg/mL. Compounds **89**–**92** also exhibited potent to average activity against the L5178Y cell lines, with EC_50_ values ranging between 0.1 and 6.7 µg/mL [53].

### 2.3. Compounds Produced by Hyphomycetes

New isocoumarin derivatives, aspergisocoumrins A (**94**) and B (**95**) (Figure 6) were obtained from the culture of the endophytic fungus *Aspergillus* sp. HN15-5D derived from the fresh leaves of the mangrove plant *Acanthus ilicifolius* collected from Dongzhaigang Mangrove National Nature Reserve on Hainan Island, China. Compounds **94** and **95** exhibited cytotoxicity against MDA-MB-435, with IC_50_ values of 5.08 and 4.98 μM, respectively [54].

Two new 6,8(14),22-hexadehydro-5α,9α-epidioxy-3,15-dihydroxy sterols, nigerasterols A (**96**) and B (**97**) (Figure 6), were isolated from *Aspergillus niger* MA-132, an endophytic fungus associated with *Avicennia marina*. Compounds **96**–**97** displayed cytotoxicity against the HL60 cell line with an IC_50_ value of 0.30 and 1.50 µM, respectively. Compounds **96**–**97** also exhibited potent activity against the A549 cell line with IC_50_ values of 1.82 and 5.41 µM, respectively [55].

Four new quinazolinone alkaloids, namely, aniquinazolines A–D (**98**–**101**) (Figure 6), were identified from *Aspergillus nidulans* MA-143, an endophytic fungus associated with the leaves of the marine mangrove plant *Rhizophora stylosa*. Compounds **98**–**101** showed potent lethality against brine shrimp with LD_50_ values of 1.27, 2.11, 4.95 and 3.42 µM, respectively, which were stronger than that of the positive control colchicine (with the LD_50_ value of 88.4 µM) [56].

The compounds 3β,5α-Dihydroxy-(22*E*,24*R*)-ergosta-7,22-dien-6-one (**102**), 3β,5α,14α-trihydroxy-(22*E*,24*R*)-ergosta-7,22-dien-6-one (**103**), and beauvericin (**104**) (Figure 6), were extracted from *Aspergillus terreus* (No. GX7-3B), a mangrove endophytic fungus isolated from the leaves of *Rhizophora stylosa.* Compounds **102** and **104** exhibited good or moderate cytotoxic activity against MCF-7, A549, HeLa and KB cell lines with IC_50_ values of 4.98, 1.95, 0.68, 1.50 and 2.02, 0.82, 1.14, 1.10 µM, respectively; compound **103** exhibited poor activity against the cell lines tested, namely MCF-7 (25.4 µM), A549 (27.1 µM), HeLa (24.4 µM) and KB (19.4 µM) [57].

A new compound, botryosphaerin F (**105**), and a known compound, LL-Z1271β (**106**) (Figure 6), were obtained from the mangrove fungus *Aspergillus terreus* (No. GX7-3B) isolated from the branch of *Brugnieria gymnoihiza*, growing in a coastal salt marsh of the South China Sea in Guangxi Province, China. Compound **105** showed potent inhibiting activity towards the MCF-7 and HL-60 cancer cell lines with IC_50_ values of 4.49 and 3.43 µM, respectively, and compound **106** exhibited promising activity against the HL-60 cell line with an IC_50_ value of 0.6 µM [58].

A new eudesmane-type sesquiterpenoid, penicieudesmol B (**107**) (Figure 6), was isolated from the mangrove-derived endophytic fungus *Penicillium* sp. J-54 associated with the leaves of *Ceriops tagal*, which were collected in Dong Zhai Gang Mangrove Reserve in Hainan province, China. Compound **107** exhibited weak cytotoxicity against K-562 with an IC_50_ value of 90.1 µM, with paclitaxel as the positive control (IC_50_ = 9.5 µM) [59].

**Figure 7:**
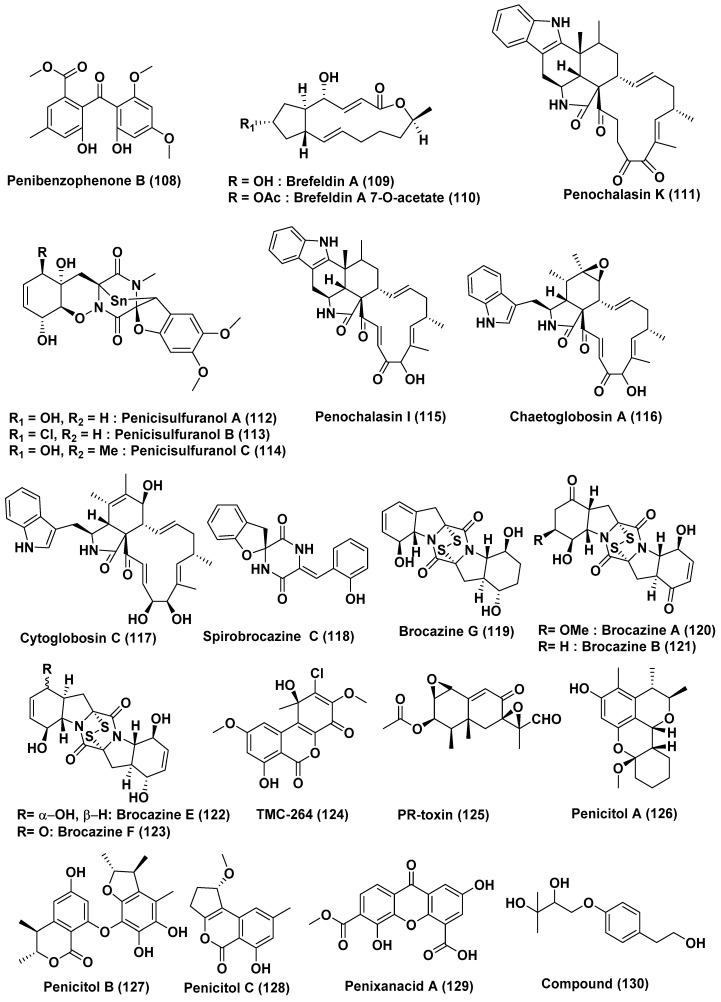
Structures of metabolites isolated from Hyphomycetes (**108**–**130**).

A new compound Penibenzophenone B (**108**) (Figure 7), was obtained from the endophytic fungus *Penicillium citrinum* HL-5126 isolated from the mangrove *Bruguiera sexangula* var. *rhynchopetala* collected in the South China Sea. The new compound **108** displayed cytotoxic activity against human A549 cell lines with an IC_50_ value of 15.7 µg/mL [60].

Five new derivatives of macrolide antibiotic Brefeldin A (**109**), along with Brefeldin A 7-*O*-acetate (**110**) (Figure 7), were produced by an endophytic fungus, *Penicillium* sp., which was isolated from the healthy root of *Panax notoginseng*. Compounds **109**–**110** exhibited cytotoxic activity against the 293, HepG2, Huh7 and KB cell line with an ID_50_ values from 0.024 to 0.62 µM. Further, studies of the cellular mechanism of compounds **109**–**110** showed that they arrested HepG2 cells at the S phase [61].

A new chaetoglobosin, penochalasin K (**111**) (Figure 7), was extracted from the mangrove endophytic fungus *Penicillium chrysogenum* V11. Its structure was elucidated by 1D, 2D NMR spectroscopic analysis and high resolution mass spectroscopic data. Compound **111** showed strong cytotoxicity against the MDA-MB-435, SGC-7901 and A549 cell lines with an IC_50_ less than 10 µM [62].

The new epipolythiodioxopiperazine alkaloids, penicisulfuranols A–C (**112**–**114**) (Figure 7), were obtained from *Penicillium janthinellum* HDN13-309, the mangrove endophytic fungus associated with *Sonneratia caseolaris* collected from Hainan Province, China. Compounds **112**–**114** showed cytotoxicity against HeLa and HL-60 cell lines, with IC_50_ values ranging from 0.1 to 3.9 µM [63].

*Penicillium chrysogenum* V11, a mangrove endophytic fungus was the source of a novel chaetoglobosin named penochalasin I (**115**), along with chaetoglobosins A (**116**), and cytoglobosin C (**117**) (Figure 7). Compound **115** exhibited marked cytotoxicity against MDA-MB-435 and SGC-7901 cells (IC_50_ < 10 µM), and compounds **116** and **117** showed potent cytotoxicity against SGC-7901 and A549 cells (IC_50_ < 10 µM) [64].

Using the one strain many compounds (OSMAC) approach, new diketopiperazines, spirobrocazine C (**118**) and brocazine G (**119**) (Figure 7) were characterized from *Penicillium brocae* MA-231, an endophytic fungus associated with *Avicennia marina* collected at Hainan Island, China. Compound **119** exhibited potent cytotoxic activity against the A2780 and A2780 CisR cell lines, with IC_50_ values of 664 and 661 nM, respectively. This activity is higher than the cisplatin where the IC_50_ values were reported as 1.67 and 12.63 µM, respectively. Compound **118** showed moderate activity against A2780 cells with an IC_50_ value of 59 µM [65]. Previously reported disulfide-bridged diketopiperazine derivatives, brocazines A (**120**), B (**121**), E (**122**), F (**123**) (Figure 7) were isolated from the same fungus. Compounds **120**–**123** displayed cytotoxic activity against the Du145, HeLa, HepG2, MCF-7, NCI-H460, SGC-7901, SW1990, SW480, and U251 cell lines with IC_50_ values ranging from 0.89 to 9.0 µM. Compounds **121** and **122** exhibited strong activity against the SW480 cell line with IC_50_ values of 2.0 and 1.2 µM, respectively, while compound **123** showed potent activity against the DU145 and NCI-H460 cell lines, with IC_50_ values of 1.7 and 0.89 µM, respectively [66].

The fungal metabolites TMC-264 (**124**), and PR-toxin (**125**) (Figure 7) were obtained from *Penicillium chermesinum* strain HLit-ROR2, an endophytic fungus associated with the root of *Heritiera littoralis*, collected from Samut Sakhon province, Thailand. Compound **124** showed cytotoxicity against T47D and MDA-MB231 cell lines with the IC_50_ value of 1.08 and 2.81 µM, respectively, while a positive control, doxorubicin, showed activity with IC_50_ of 1.55 and 2.24 µM respectively. Compound **124** also showed cytotoxic activity against the HepG2 cell line, with an IC_50_ value of 3.27 µM, which was 11 times more potent than that of etoposide, an anticancer drug (IC_50_ 35.66 µM). Besides, compound **124** selectively exhibited cytotoxic activity toward MOLT-3 and T47D cancer cells with the IC_50_ values of 1.36 and 1.08 µM and with selectivity index (SI) values of 9 and 11, respectively (IC_50_ 12.64 µM for the normal cell line, MRC-5). Compound **125** exhibited cytotoxicity against the HuCCA-1, HeLa, T47D, and MDA-MB231 cell lines with IC_50_ values in the range of 0.81–2.19 µM, which was comparable to that of doxorubicin (IC_50_ 0.26–2.24 µM). Interestingly, the compound **125** exhibited cytotoxicity against the HL-60 cell line with an IC_50_ value of 0.06 µM, which is superior to doxorubicin (IC_50_ 1.21 µM). Compound **125** selectively showed cytotoxic activity against MOLT-3 and HL-60 cancer cell lines with IC_50_ of 0.09 and 0.06 µM, respectively, with respective SI values of 40 and 61 (IC_50_ 3.66 µM for the MRC-5 cell line) [67].

Three new citrinin analogs, penicitols A–C (**126**–**128**), and one new xanthone derivative, penixanacid A (**129**) (Figure 7), were isolated from *Penicillium chrysogenum* HND11–24 isolated from the rhizosphere soil of *Acanthus ilicifolius*. Compounds **126**–**127** showed good activity against the HeLa, BEL-7402, HEK-293, HCT-116, and A549 cell lines with IC_50_ values of 4.6−10.5 and 3.4−9.6 µM, respectively. Compounds **128**–**129** exhibited cytotoxicity against the HeLa, BEL-7402, HEK-293, HCT-116, and A549 cell lines with IC_50_ values of 10–40.5 µM [68].

**Figure 8:**
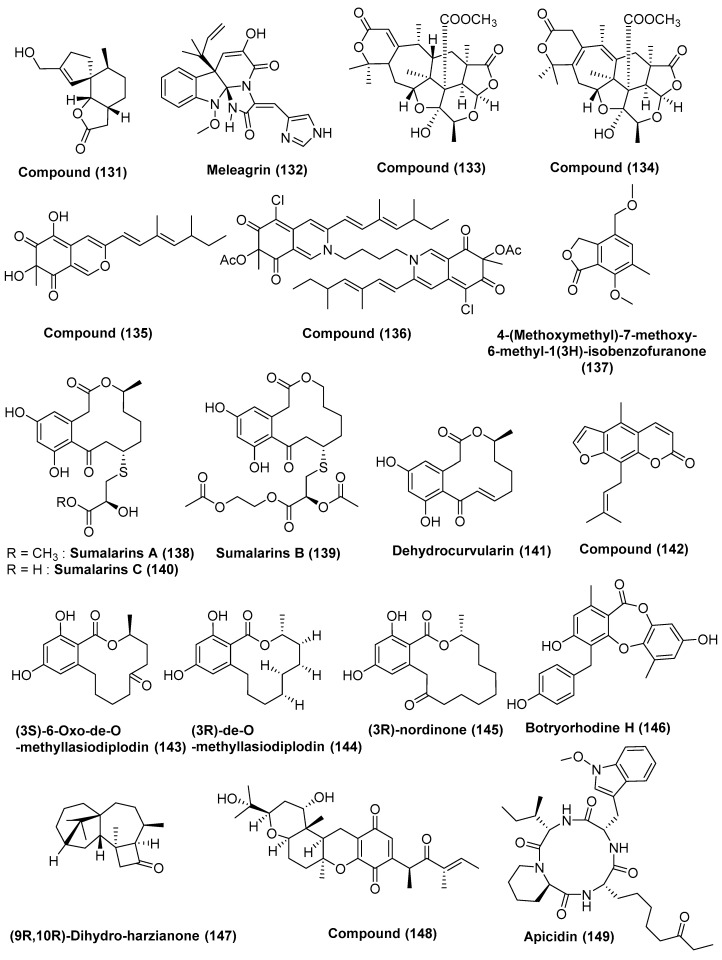
Structures of metabolites isolated from Hyphomycetes (**131**–**149**).

Two new metabolites, compounds **130** (Figure 7) and **131** (Figure 8), were obtained from *Penicillium* sp. FJ-1, associated with *Avicennia marina* collected in Fujian, China. Compound **131** exhibited anti-proliferative activity against Tca8113, MG-63 and the normal liver cell line WRL-68 with an IC_50_ value of 10 µM, 55 nM and 58 µM, respectively. Compound **131** has also shown a significant inhibition of tumor growth of human osteosarcoma when tested against nude mice. Compound **130** exhibited weak activity against Tca8113 and MG-63 cells with the IC_50_ values of 26 µM and 35 µM, respectively, in the anti-proliferative assay. Taxol, the positive control, showed activity against Tca8113 and MG-63 cell lines with IC_50_ values of 46 nM and 10 nM, respectively [69].

Meleagrin (**132**) (Figure 8) was isolated from the endophytic fungus *Penicillium* sp. GD6, associated with the Chinese mangrove *Bruguiera gymnorrhiza* collected off the coasts of Zhanjiang, China. Compound **132** showed potent cytotoxic activity against two tumor cell lines, HL60 and A549, with IC_50_ values of 9.7 and 8.3 µM, respectively [70].

Mangrove endophytic fungus, *Penicillium* 303# obtained from the sea water in Zhanjiang Mangrove National Nature Reserve in Guangdong Province, China, was the source of compounds **133**, **134**, **135** and **136** (Figure 8). Compounds **133**–**135** exhibited cytotoxicity against MDA-MB-435, HepG2, HCT-116, and A549 with IC_50_ value of 11.9–37.82 µg/mL. Compound **136** showed strong cytotoxic activity against the MDA-MB-435 cell lines with IC_50_ values of 7.13 µM and average to low activity against the HepG2 and HCT-116 cell lines with IC_50_ values of 39.64 and 27.80 µM, respectively [71].

A new isobenzofuranone, 4-(methoxymethyl)-7-methoxy-6-methyl-1(3*H*)-isobenzofuranone (**137**) (Figure 8), was isolated from the mangrove endophytic fungus, *Penicillium* sp. ZH58, which was associated with the leaves of mangrove tree *Avicennia* from Dong Sai, Hainan, on the South China Sea coast. Compound **137** showed cytotoxic activity against the KB and KB_V_200 cell lines with IC_50_ values of 6 and 10 µg/mL, respectively [72].

The new and rare sulfur-containing curvularin derivatives sumalarins A, B, (**138**, **139**) C (**140**) (Figure 8), and dehydrocurvularin (**141**) (Figure 8) were obtained from *Penicillium sumatrense* MA-92, isolated from the rhizosphere of the mangrove *Lumnitzera racemose* collected at Wen Chang on Hainan Island, China. Compounds **138**–**141** displayed cytotoxic activities against the Du145, HeLa, Huh 7, MCF-7, NCI-H460, SGC-7901, and SW1990 cell lines, with IC_50_ values in the range of 3.8 to 10 µM [73].

*Penicillium* sp. ZH16, a mangrove endophytic fungus, obtained from the South China Sea, was the source of the furanocoumarin 5-methyl-8-(3-methylbut-2-enyl) furanocoumarin (**142**) (Figure 8). Compound **142** exhibited cytotoxicity against the KB and KB_V_200 cell lines with IC_50_ values of 5 and 10 µg/mL, respectively [74].

Zhang et al. [75] reported the production of the compounds (3*S*)-6-oxo-de-*O*-methyllasiodiplodin (**143**), (3*R*)-de-*O*-methyllasiodiplodin (**144**), (3*R*)-nordinone (**145**) (Figure 8), by the co-culturing of mangrove endophytic fungus *Trichoderma* sp. 307 isolated from the stem bark of *Clerodendrum inerme*, and the aquatic pathogenic bacterium *Acinetobacter johnsonii* B2 isolated from the stem bark of *Clerodendrum inerme*, collected in Zhanjiang Mangrove National Nature Reserve in Guangdong Province, China. Compound **144** exhibited good cytotoxic activity against the GH3 and MMQ cell lines with IC_50_ values of 6.44 and 6.58 µM, respectively, and against rat normal pituitary cells (RPC) with an IC_50_ value of 6.94 µM. Compound **145** displayed average cytotoxic activity against the GH3 and MMQ cell lines with IC_50_ values of 12.33 and 10.13 µM, respectively, and against RPC cell lines with an IC_50_ value of 100.03 µM. Compound **143** was less active against the GH3 and MMQ cell lines with IC_50_ values of 21.42 and 13.59 µM, respectively, and against the RPC cell lines with an IC_50_ value of 142.8 µM as a positive control [75].

Zhang et al. [76] reported the production of a new depsidone, botryorhodine H (**146**) (Figure 8), by co-culturing mangrove endophytic fungus *Trichoderma* sp. 307 and *Acinetobacter johnsonii* B2. Compound **146** exhibited good cytotoxic activity against the MMQ and GH3 cell lines with IC_50_ values of 3.09 and 3.64 µM, respectively.

A new harziane diterpenoid, named (9*R*,10*R*)-dihydro-harzianone (**147**) (Figure 8), was isolated from *Trichoderma* sp. Xy24, an endophytic fungus residing in the leaves, stems, and peels of the mangrove plant *Xylocarpus granatum*, collected in the Sanya district of Hainan province, China. Compound **147** was active against the HeLa and MCF-7 cell lines with IC_50_ values of 30.1 µM and 30.7 µM, respectively [77].

The endophytic fungus *Nigrospora* sp. MA75 associated with *Pongamia pinnata*, which led to the isolation of a new compound 2,3-didehydro-19a-hydroxy-14-epicochlioquinone B (**148**) (Figure 8) when grown in medium containing 3.5% NaI. Compound **148** showed potent cytotoxicity against the MCF-7, SW1990, and SMMC7721 cell lines, with IC_50_ values of 4, 5, and 7 µg/mL, respectively [78].

A known cyclic peptide, beauvericin (**104**) (Figure 6), was obtained from *Fusarium* sp. (No. DZ27) an endophytic fungus residing inside the bark of *Kandelia candel* from Dongzhai mangrove, Hainan, China, in the South China Sea. Compound (**104**) showed cytotoxic activity against the KB and KBv200 cell lines with IC_50_ values of 5.76 and 5.34 µM, respectively. It induces apoptosis through the mitochondrial pathway, including the decrease of relative oxygen species generation, the loss of mitochondrial membrane potential, the release of cytochrome c, the activation of Caspase-9 and -3, and the cleavage of PARP. Additionally, the regulation of Bcl-2 or Bax was not involved in the apoptosis induced by beauvericin in KB and KBv200 cells [79].

An inhibitor of histone deacetylase, Apicidin (**149**) (Figure 8), was isolated from *Fusarium* sp., an endophytic fungus associated with the leaf of mangrove *Kandelia candel* planted at Dongzhai Harbor on Hainan Island, China. Apicidin showed good cytotoxic activity against GLC-82 cells with the IC_50_ value of 6.94 ± 0.27 µM. Apicidin suppressed proliferation and invasion, and induced apoptosis via the mitochondrial pathway in GLC-82 cells, including the loss of ΔΨm, the release of cytochrome c from mitochondria, the activation of caspase-9 and -3, and the cleavage of poly-ADP-ribose polymerase [80]. Apicidin **149**, was previously isolated from the mangrove endophytic fungus ZZF42 from the South China Sea and exhibited selective in vitro cytotoxicity towards KB and KBv200 with IC_50_ values of less than 0.78 µg/mL [81].

**Figure 9:**
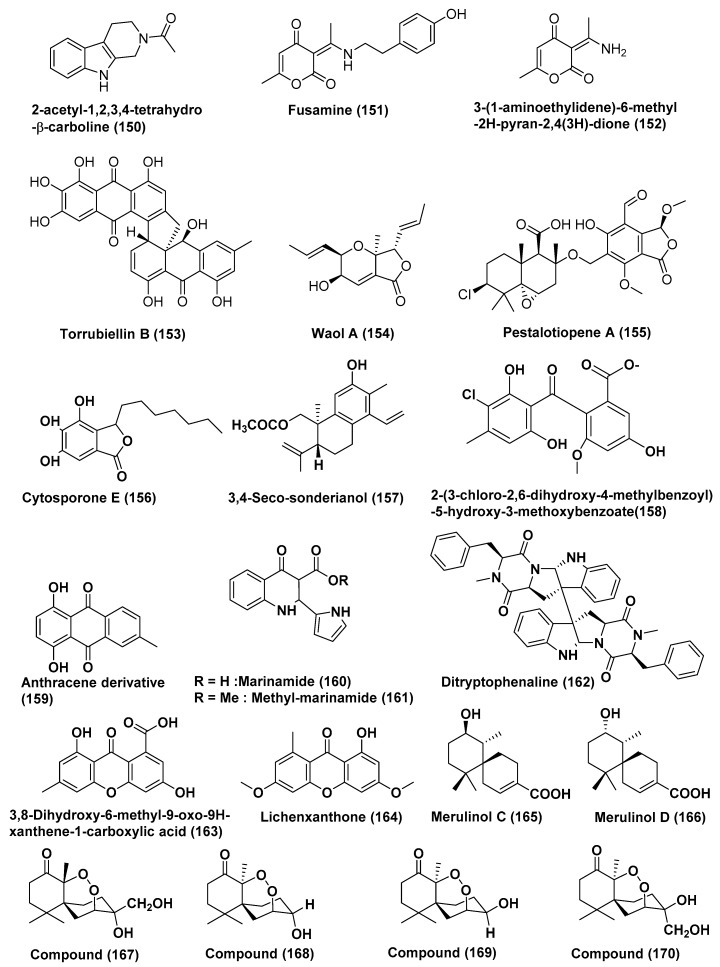
Structures of metabolites isolated from Hyphomycetes (**150**–**170**)*.*

An unusual alkaloid 2-acetyl-1,2,3,4-tetrahydro-β-carboline (**150**), fusamine (**151**), and 3-(1-aminoethylidene)-6-methyl-2*H*-pyran-2,4(3*H*)-dione (**152**) (Figure 9), were isolated from the culture broth of *Fusarium incarnatum* (HKI0504), an endophytic fungus of the mangrove plant *Aegiceras corniculatum*. Compound **150** exhibite weak antiproliferative activity against HUVEC and the K-562 cell line with GI_50_ values of 41.1 and 33.3, respectively. Compounds **151** and **152** exhibit anti-proliferative activity against HUVEC and the GI50 K-562 cell line with GI_50_ values of 37.3, 37.6, and 41.1, 33.3, respectively. The compounds **150**, **151** and **152** displayed cytotoxic activity against HeLa cells with a CC_50_ value of 23.8, 23.3, and 23.8 µM, respectively. The standard imatinib exhibited anti-proliferative activity against HUVEC and the GI50 K-562 cell line with a GI_50_ value of 18.5 and 0.17, respectively, and cytotoxic activity against HeLa cells with a CC_50_ value of 65.8 µM [82].

*Acremonium* sp., an endophytic fungus residing inside the leaves of *Sonneratia caseolaris* collected from Hainan, China, was the source of a dimeric anthracene derivative torrubiellin B (**153**) (Figure 9). Compound **153** exhibited potent cytotoxic activity against the cisplatin-sensitive cell lines Cal27, Kyse510, HCC38, A2780, MDA-MB-231with IC_50_ values ranging from of 0.3 to 1.5 µM, and against the cisplatin-resistant cell lines Cal27, Kyse510, HCC38, A2780, MDA-MB-231 with IC_50_ values ranging from 0.2 to 2.6 µM. The positive control cisplatin exhibited cytotoxic activity against the tested cell lines with IC_50_ values in the range of 1.5–38.1 µM [83].

Waol A (**154**), pestalotiopene A (**155**), and cytosporone E (**156**) (Figure 9) were obtained from the endophytic fungus *Acremonium strictum*, isolated from the mangrove tree *Rhizophora apiculate*, collected on the island of CatBa, Vietnam. Compounds **154**–**156** showed moderate cytotoxic activity against human cisplatin-sensitive (IC_50_ values 27.1, 76.2, and 8.3 µM, respectively) and resistant A2780 cell lines (IC_50_ values 12.6, 30.1, and 19.0 µM, respectively) [84].

The compound 3,4-seco-sonderianol (**157**) (Figure 9), a known diterpenoid, was obtained from the endophytic fungus J3 of *Ceriops tagal*, collected in Hainan province, China. Compound **157** showed cytotoxic activity against the K562, SGC-7901, and BEL-7402 cell lines with IC_50_ values of 9.2, 15.7, and 25.4 µg/mL, respectively. The positive control paclitaxel displayed activity against the K562, SGC-7901 and BEL-7402 cell lines with IC_50_ values of 5.1, 1.6 and 6.3 µg/mL, respectively [85].

The compound 2-(3-chloro-2,6-dihydroxy-4-methylbenzoyl)-5-hydroxy-3-methoxybenzoate (**158**) (Figure 9), was extracted from the endophytic fungus No. ZH-3 from the South China Sea. Compound **158** showed cytotoxic activity against the hepG2 cell line (IC_50_ = 25 µg/mL) [86].

Mangrove endophytic fungus No.5094, which was collected in the South China Sea was the source of anthracene derivative (**159**) (Figure 9). Compound **159** exhibited potent activity towards the KB and KBv200 cell lines with LD_50_ values of 5.5 and 10.2 µM, respectively [87].

Marinamide (**160**) and methyl marinamide (**161**) (Figure 9) were obtained by co-cultures of two marine-derived mangrove endophytic fungi (strains Nos. 1924 and 3893) from the South China Sea coast. Their structures were elucidated using comprehensive spectra methods. Compound **160** was found to be cytotoxic with IC_50_ values of 7.0, 0.4, 91 nM and 0.529 µM, respectively against HepG2, 95-D, MGC832 and HeLa cells. For the similar cell lines, compound **161** exhibited cytotoxicity with IC_50_ values of 2.52, 1.54, 13.0 and 0.110 µM, respectively [88].

Mangrove endophytic fungus No. Gx-3a in the South China Sea was the source of ditryptophenaline (**162**) (Figure 9). Compound **162** exhibited potent cytotoxic activity against KB and KBv200 cells, with LD_50_ values of 8.0 µM and 12.0 µM [89].

The 3, 8-Dihydroxy-6-methyl-9-oxo-9*H*-xanthene-1-carboxylate (**163**) and lichenxanthone (**164**) (Figure 9), were isolated from the mangrove endophytic fungus No·SK7RN3G_1_ from the South China Sea. Compounds **163**–**164** exhibited cytotoxicity against the HepG2 cell line with IC_50_ values of 20 and 25 µg/mL, respectively [90].

### 2.4. Compounds Produced by Basidiomycetes

Two new chamigrane sesquiterpenes, merulinols C and D (**165**, **166**) (Figure 9), were isolated from the culture of the basidiomycetous fungus XG8D, isolated from the healthy leaves of *Xylocarpus granatum*, collected in Samutsakorn province, Thailand. Compounds **165** and **166** selectively displayed cytotoxicity against KATO-3 cells with IC_50_ values of 35.0 and 25.3 µM, respectively [91].

Compounds **167**, **168**, **169** and **170** (Figure 9) were extracted from *Pseudolagarobasidium acaciicola*, associated with *Bruguiera gymnorrhiza* from Samut Sakhon province, Thailand. These compounds were evaluated for their cytotoxicity against the HuCCA-1, A549, MOLT-3, HepG2, HL-60, MDA-MB231, T47D, and HeLa cancer cell lines and normal human embryonic lung cell lines (MRC-5). Compound **167** displayed cytotoxicity against the HuCCA-1, A549, MOLT-3, HepG2, HL-60, MDA-MB231, and T47D cancer cell lines with IC_50_ values in the range of 0.28–37.46 µM and against the MRC-5 normal cell line with an IC_50_ value of 17.92 µM. Compound **167** selectively showed activity against the HL-60 cell line with an IC_50_ value of 0.28 µM and a selectivity index (SI) value of 64.0. Compound **168** showed activity against the A549, MOLT-3, HepG2, HL-60, MDA-MB231, T47D, HeLa and MRC-5 cells IC_50_ values in the range of 12.09–170.08 µM. Compounds **169** and **170** were active against the cell lines tested, IC_50_ 15.20–76.97 µM for **169** and IC_50_ 18.31–154.51 µM for **170**. Compound **170** also selectively exhibited cytotoxic activity toward the HL-60 cell line (IC_50_ 18.31 µM) with an SI value of 4.4 [92].

**Figure 10:**
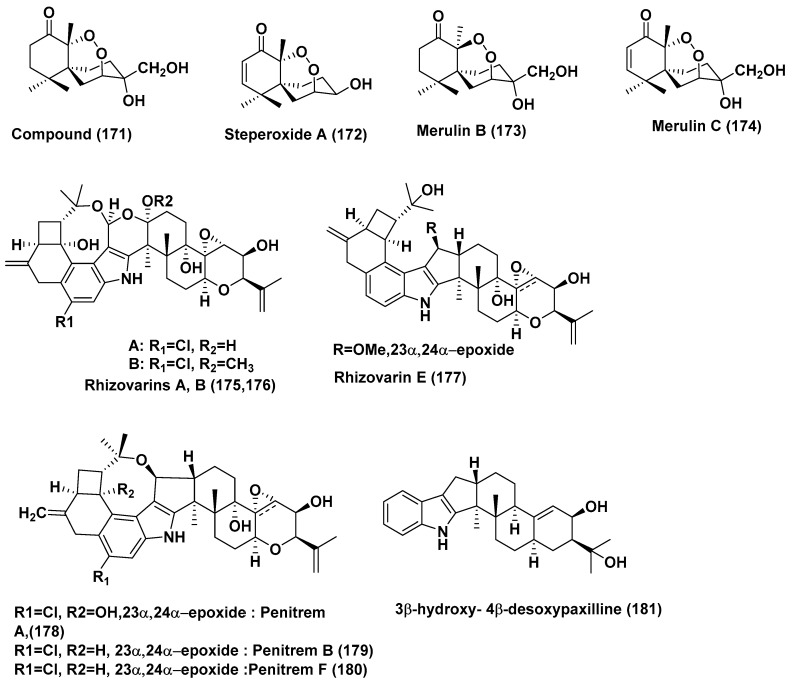
Structures of metabolites isolated from Basidiomycetes (**171**–**174**) and from Zygomycetes (**175**–**181**).

A new nor-chamigrane endoperoxide, 3*-epi*-Steperoxide A (**171**), along with the known sesquiterpenes steperoxide A (**172**), merulin B (**173**), and merulin C (**174**) (Figure 10) were isolated from *Pseudolagarobasidium acaciicola*, an endophyte residing inside the mangrove plant *Bruguiera gymnorrhiza*. Compounds **171** and **172** showed strong cytotoxic activity with IC_50_ ranges of 0.68–3.71 and 0.67–5.25 µg/mL, respectively, against MOLT-3, HuCCA-1, A549, HepG2, HL-60, MDA-MB-231, T47D, and HeLa cells. Compound **173** exhibited weak activity against MOLT-3, A549, HepG2, HL-60, MDA-MB-231 and T47D cells with an IC_50_ ranging from 11.94–49.08 µg/mL, but was inactive toward HuCCA-1 and HeLa cells at 50 µg/mL. Compound **174** showed the most potent cytotoxic activity against HL60 cancer cells, with an IC_50_ value of 0.08 µg/mL, whereas it displayed activity toward the MOLT-3, HuCCA-1, A549, HepG2, MDA-MB-231, T47D, and HeLa cell lines with an IC_50_ range of 0.19–3.75 µg/mL [93].

### 2.5. Compounds Produced by Zygomycetes

The rhizovarins A, B, E (**175**–**177**), penitrems A, C, F (**178**–**180**) and 3β-hydroxy-4β-desoxypaxilline (**181**) (Figure 10) were obtained from *Mucor irregularis* QEN-189, an endophytic fungus residing inside the inner tissue of *Rhizophora stylosa*, collected in Hainan Island, China. Compounds **175**–**181** showed cytotoxicity against the human A-549 cell lines with IC_50_ values of 11.5, 6.3, 9.2, 8.4, 8.0, 8.2, and 4.6 µM, while compounds **175**, **176**, **178**–**181** were cytotoxic against the human HL-60 cell lines with IC_50_ values of 9.6, 5.0, 7.0, 4.7, 3.3 and 2.6 µM, respectively. Adriamycin, a positive control, exhibited activity against A-549 and HL-60 cell lines with IC_50_ values of 0.30 and 0.06 µM, respectively [94].

## 3. Methods Used for the Activation of Silent Biosynthetic Genes

Recent studies in the marine-based microorganisms have shown that these microorganisms are a rich source for novel bioactive compounds. Salinosporamide A (marizomib), a microbial compound isolated from marine *Salinispora* bacteria with proteasome inhibitory activity is expected to be a future anti-cancer drug, and is presently under clinical trials [95]. However, the reoccurrence of the same compound as discovered in terrestrial sources, in marine microorganisms often leads to serious issues. Advances in molecular biology have enhanced our understanding regarding how to exploit the genetic potential of bacteria and fungi to produce newer chemical entities apart from those that are currently known, which have yet to be explored [96,97]. It has been reported that under laboratory conditions, biosynthetic genes are not expressed as such, as only limited bioactive compounds are produced by these microbes. To overcome these limitations, different strategies have been proposed, including culturing promising strains in varying culture media and under a variety of culture conditions [98], mixing cultures of two or more microbe variants and epigenetic modifications that treat microbes with epigenetic modifiers such as histone deacetylase inhibitors or DNA methyl transferase to initiate the transcription of silent genes [99,100] to enhance the variation and diversity of the produced metabolites.

### 3.1. The Co-Culture Strategy

Microbes in natural ecosystem conditions always harbor and flourish in co-existence with a variety of microbes. Antagonism and competition for limited resources often lead to high competition among species, and microbes adopt various defense strategies, which favor the production of important bioactive secondary metabolites [101]. The co-culturing of two or more different microbes at the laboratory scale might mimic the ecological setting and induce the cascade of genes responsible for biosynthesis that are normally are masked under optimum culture parameters. Co-cultivation of two *Aspergillus* species derived from mangroves produced the new alkaloid aspergicin and the previously recognized compounds neoaspergillic acid and ergosterol, with antibacterial activity [102]. Li et al. [103] co-cultured two mangrove epiphytes and identified a novel xanthone derivative compound that showed antifungal activity. Two new alkaloids, marinamide, and marinamide methylether, were reported from mangrove-derived endophytic fungi with a cytotoxic effect when grown in mixed fermentation [88]. Pestalone, a chlorinated prenylsecoanthraquinone, was produced by the marine-derived fungus *Pestalotia* sp. when grown in the presence of the marine-derived bacterium *Thalassopia* sp., which belongs to the Gram-negative group. [104]. When *Libertella* sp., a marine-based fungi, were cultured in the presence of the bacteria *Thalassopia* sp., it resulted in the production of diterpenoid libertellenones of fungal origin [105]. In another set of studies, when the bacterium *Sphingomonas* sp. was grown in the presence of *Aspergillus fumigatus*, a novel compound glionitrin A, a diketopiperazine disulfide, was identified and appeared to show strong cytotoxicity against HCT-116, A549, AGS and DU145 cells [106]. These studies suggest that co-cultivation has tremendous potential to generate novel chemical entities from microbes when cultured under laboratory conditions.

### 3.2. Epigenetic Modification

The addition of epigenetic modifiers to fungi would allow us to induce cryptic fungal gene clusters. This technique can be applied to any fungal strain and does not require strain-dependent genetic manipulation. Williams et al. [107] reported that epigenetic modifiers could be rationally employed to access silent natural product pathways. Histone deacetylase (HDAC) or DNA methyltransferase (DMAT) are often used as epigenetic agents to change the transcription rate of some genes [108]. Henrikson et al. [12] reported the identification of nygerone A from *A. niger* when grown with suberoylanilide hydoxamic acid (SAHA). Wang et al. [109] reported induced metabolite generation in *Penicillium citreonigrum* when grown in the presence of methyl transferase inhibitor, 5-azacytidine (5-AZA). When *Hypoxylon* sp., an endophytic fungi, was treated with the epigenetic modifiers SAHA and AZA it enhanced the production of volatile organic compounds (VOCs) [110]. The marine endophytic fungus *Leucostoma persoonii* from *Rhizophora mangle* enhanced the production of cytosporones B, C, E and R in HDAC inhibited fermentation [111]. These studies provide evidence that the use of epigenetic modifiers modulate secondary metabolite production, resulting in different gene expressions.

## 4. Conclusions

Mangrove fungi are a ubiquitous source of novel bioactive metabolites with the potential to display anticancer properties. It is interesting to observe the chemical diversity in these metabolites, which include simple glycoside (**27**) and peptide molecules (pullularins E, **89**; F, **90** and apicidin, **149**) as well as complex stereospecific structures such as cytochalasin H (**18**), phomopsichalasin G (**19**), aniquinazolines A–D (**98**–**101**) and penitrem A, B and F (**178**–**180**). Chemical diversity plays an important role in the drug discovery pipeline, as this provides structurally diverse scaffolds that display similar activity via different modes and/or mechanisms of action. This phenomenon is also observed in mangrove fungal metabolites, as they show potent anticancer activity via different mechanisms of action such as apoptotic cell death (SZ-685C, **73**; beauvericin, **104**), the inhibition of kinase proteins involved in signal transduction pathways (Mycoepoxydiene, **32**; Altersolanol A, **64**; and the inhibition of topoisomerase I (**36**). Although many metabolites demonstrated moderate cytotoxic activities against cancer cell lines, only a few displayed superior activity than the standard anticancer drugs (**98**–**101**, **119**, **124**). It can be suggested that the rational derivatization of metabolites may provide molecules with better activity against a wide range of cancer cell lines. In addition, the identified metabolites with broad-spectrum anticancer activity need to be investigated to establish their mechanisms of action and to develop as novel anticancer therapeutics.

## Figures and Tables

**Table 1:** Novel anticancer bioactive compounds reported from mangrove fungi.

Sr. No.	Fungus	Host Plant(s)	Plant Part or Tissue Locality of Host Plants	Compounds Isolated	Cell Line	IC_50/_EC_50_/Inhibition	Refs.
Compounds Produced by *Coelomycetes*							
**1**	*Pestalotiopsis* sp.	*Rhizophora mucronata*	Not reported	Demethylincisterol A3 (**1**)	HeLa, A549 and HepG	In the range of 0.17 to 14.16 nM	[16]
				Ergosta-5,7,22-trien-3-ol (**2**), stigmastan-3-one (**3**), stigmast-4-en-3-one (**4**), stigmast4-en-6-ol-3-one (**5**), flufuran (**6**)	HeLa, A549 and HepG	In the range of 11.44–102.11 µM	
**2**	*Pestalotiopsis microspore*	*Drepanocarpus lunatus*	Cameroon	Compound (**7**) Pestalotioprolide D–F (**8**–**10**)	L5178Y	0.7, 5.6, 3.4, and 3.9 µM	[17]
				Pestalotioprolide E (**9**)	A2780	1.2 µM	
**3**	*Pestalotiopsis clavispora*	*Rhizophora harrisonii*	Port Harcourt, Nigeria	Pestalpolyol I (**11**)	L5178Y	4.10 µM	[18]
**4**	*Pestalotiopsis vaccinia*	*Kandelia candel*	China	Pestalamine A (**12**)	MCF-7, HeLa, and HepG2	40.3, 22.0, and 32.8 µM	[19]
**5**	*Phoma* sp. OUCMDZ-1847	Fruit sample of *Kandelia candel*	Wenchang, Hainan Province, China	Phomazines B (**13**), epicorazine A (**14**), epicorazine B (**15**), epicorazine C (**16**), exserohilone A (**17**)	HL-60, HCT-116, K562, MGC-803, and A549 Cells	In the range 0.05 to 8.5 µM	[20]
**6**	*Phomopsis* sp.		Zhanjiang, China	Cytochalasin H (**18**)	A549 cells	Arrested A549 cells at the G2/M phase, inhibited the migration ability of A549 cells in a dose-dependent manner	[21]
**7**	*Phomopsis* spp. xy21 and xy22	Leaves *Xylocarpus granatum*	Trang Province, Thailand	Phomopsichalasin G (**19**)	HCT-8, HCT-8/T, A549, MDA-MB-231, and A2780 cancer Cells	7.5, 8.6, 6.4, 3.4, and 7.1 µM	[22]
**8**	*Phomopsis longicolla* HL-2232	*Bruguiera sexangula* var. *rhynchopetala*		6-aminopurine-9-carboxylic acid Me ester (**20**), uridine (**21**)	B16F10, A549, HL-60 and MCF-7 Cells	14.9 and 8.6 µM	[23]
**9**	*Phomopsis* sp. HNY29-2B	Branch of *Acanthus llicifolius*	South China Sea, Hainan province, China	Dicerandrol A (**22**)	MDA-MB-435, HCT-116, Calu-3 and Huh7 Cells	3.03, 2.64, 1.76 and 4.19 µM	[24]
				Dicerandrol B (**23**), Penexanthone A (**26**)	MDA-MB-435, HCT-116 and Calu-3	<10 µM	
				Dicerandrol C (**24**)	MDA-MB-435, HCT-116, Calu-3, MCF-10A Cells	44.10, 42.63, 36.52, and 33.05 µM	
				diacetyl phomoxanthone B (**25**)	MDA-MB-435, HCT-116, Calu-3, Huh7 Cells	14.40, 7.12, 4.14 and 29.20 µM	
**10**	*Phomopsis* sp. (ZH76)	*Excoecaria agallocha*	Dong Sai, South China Sea coast	3-*O*-(6-*O*-α-l-arabinopyranosyl)-β-d-glucopyranosyl-1,4-dimethoxyxanthone (**27**)	HEp-2 and HepG2	9 and 16 µM	[25]
**11**	*Diaporthe* sp. SCSIO 41011	*Rhizophora stylosa*	Sanya city, Hainan Province, China	Isochromophilone D (**28**)	786-O cells	8.9 µM	[26]
				epi-Isochromophilone II (**29**)	ACHN, OS-RC-2, and 786-O cells,	In the range of 3.0 to 4.4 µM, Sorafenib (3.4 to 7.0 µM)	
**12**	*Diaporthe phaseolorum* SKS019	Branches of *Acanthus ilicifolius*	Shankou, Guangxi province, China	5-deoxybostrycoidin (**30**)	MDA-MB-435 and NCI-H460 cancer cells	5.32 and 6.57 µM	[27]
				Fusaristatin A (**31**)	MDA-MB-435 cancer cells	8.15 µM	
**13**	*Phomosis* sp. A818	Foliage of *Kandelia candel*	Fujian Province, China	Mycoepoxydiene (**32**), deacetylmycoepoxydiene (**33**)	MDA-MB-435	7.85 and 14.61 µM,	[28]
**14**	*Phomosis* sp. A818	Foliage of *Kandelia candel*	Fujian Province, China	Mycoepoxydiene (**32**)	Suppress antigen-stimulated degranulation and cytokine production in mast cells and IgE-mediated passive cutaneous anaphylaxis in mice		[29]
Compounds Produced by *Ascomycetes*							
**15**	*Sarocladium kiliense* HDN11-84	rhizosphere soil of *Thespesia populnea*,	Guangxi Province, China	Saroclazine B (**34**)	HeLa Cells	4.2 µM	[30]
**16**	*Annulohypoxylon* sp.	*Rhizophora racemosa*	Cameroon	Daldinone I (**35**)	Ramos and Jurkat J16	6.6 and 14.1 µM, Potently blocks autophagy, a potential pro-survival pathway for cancer cells	[31]
**17**	*Eurotium rubrum*	*Suaeda salsa*	“BoHai” seaside, China	Rubrumol (**36**)	A549, MDA-MB-231, PANC-1 and HepG2	Cytotoxic	[32]
					Topo I	Relaxation activity The band backward shifting and trailing of rubrumol (36) was observed at 100, 50, 10, 5 and 1 µM	
**18**	*Eutypella* sp. 1–15	Soil of mangrove rhizosphere in Jimei, Fujian Province, China	Not reported	13-Hydroxy-3,8,7(11)-eudesmatrien-12,8-olide (**37**)	JEKO-1 and HepG2	8.4 and 28.5 µM	[33]
**19**	*Rhytidhysteron rufulum* AS21B	Leaves of *Azima sarmentosa*	Samutsakhon province, Thailand	Rhytidenones G (**38**), H (**39**)), deoxypreussomerin B (**40**), palmarumycin CP17 (**41**), 1-oxo-1,4-dihydronapthalene-4-spiro-20-naptho[400-hydroxy-100,800-de][10,30]-dioxine (**42**), preussomerin EG4 (**43**), rhytidenone E (**44**), rhytidenone F (**45**), palmarumycin C5 (**46**), and 4,8-dihydroxy-3,4-dihydronaphthalen-1(2*H*)-one (**47**)	Ramos lymphoma	17.98, 0.018, 18.00, 33.1, 15, 82.9, 0.461, 0.048, 31.7 and 23.1 µM, (Ibrutinib 28.7 µM)	[34]
				Compounds (**38**), (**39**), (**44**), (**45**), and (**47**)	H1975 Cell	7.3, 0.252, 10.24, 1.17 and 50 µM (afatinib 1.97 µM)	
**20**	*Lasiodiplodia* sp. 318#	*Excoecaria agallocha*	Guangdong Province, China	2,4-Dihydroxy-6-nonylbenzoate (**48**)	MMQ and GH3 Cells	5.2 and 13.0 µM	[35]
**21**	*Lasiodiplodia* sp. 318#	*Excoecaria agallocha*	Guangdong Province, China	Ethyl-2,4-dihydroxy-6-(80-hydroxynonyl)-benzoate (**49**)	MDA-MB-435, HepG2, HCT-116, A549 and leukaemia THP1 Cells	0.13, 12.50, 11.92, 13.31 and 39.74 µM	[36]
**22**	*Lasiodiplodia theobromae* ZJ-HQ1	*Acanthus ilicifolius*	Guangdong Province, China	Chloropreussomerins A and B (**50**, **51**) Preussomerin D (**56**)	A549 and MCF-7	In the range of 5.9–8.9 µM	[37]
				Preussomerin K (**52**), Preussomerin H (**53**), Preussomerin G (**54**), Preussomerin F (**55**),	A549, HepG2, MCF-7	In the range of 2.5–9.4 µM	
**23**	*Rhytidhysteron rufulum* BG2-Y	Leaves of *Bruguiera gymnorrhiza*	Pak Nam Pran, Prachuab Kiri Khan Province, Thailand	Rhytidchromone A (**57**), B (**58**), C (**59**), and E (**60**)	Kato-3 Cells	In the range of 16.0–23.3 µM	[38]
					MCF-7 cells	19.3–17.7 µM	
**24**	*Campylocarpon* sp. HDN13-307	Root of *Sonneratia caseolaris*	China	Campyridone D (**61**), and ilicicolin H (**62**)	HeLa	8.8 and 4.7 µM	[39]
**25**	*Stemphylium globuliferum*	*Avicennia marina*	Hurghada, Egypt	Dihydroaltersolanol C (**63**), Altersolanol A (**64**), Altersolanol B (**65**), Alterporriol E (**67**)	L5178Y	3.4, 2.53, 3.78 and 6.9 µM	[40,41,42]
				Altersolanol N (**66**)	L5178Y	Low micromolar range (% growth-1.4)	
**26**	*Paradictyoarthrinium diffractum* BCC 8704	Associated with mangrove wood	Laem SonNational Park, Ranong Province, Thailand	Paradictyoarthrins A (**68**)	KB, MCF-7, NCI-H187, Vero Cells, KB, MCF-7, NCI-H187, Vero Cells	In the range of 23–31 µg/mL	[46]
				Paradictyoarthrin B (**69**)	KB, MCF-7, NCI-H187, Vero Cells	3.1, 3.8, 9.5, and 5.6 µg/mL	
				Preussomerin C (**70**), ymf 1029C (**71**) and altenusin (**72**)	KB, MCF-7, NCI-H187, Vero Cells	Moderate to poor activity	
				ymf 1029C (**71**)	NCI-H187 cells	5.0 µg/mL	
**27**	*Halorosellinia* sp. (No. 1403)	--	South China Sea	SZ-685C (**73**)	NFPA, MMQ and RPC cells	18.76, 14.51, and 56.09 µM	[47]
**28**	*Dothiorella* sp.	*Aegiceras corniculatum*	Fujian Province, China	Dothiorelone F (**74**), Dothiorelone G (**75**)	Raji cancer	2 µg/mL	[49]
**29**	*Rhytidhysteron* sp.	Leaves of *Azima sarmentosa*	Samutsakhon province, Thailand	Rhytidones B–C (**76**, **77**), MK3018 (**78**), palmarumycin CR1 (**79**)	MCF-7 and CaSki Cells	In the range of 14.47 and 25.59 µM	[50]
				Rhytidones B (**76**)	CaSki	22.81 µM	
**30**	*Sporothrix* sp.	Bark, *Kandelia candel*	South China Sea	Sporothrin A (**80**)	Inhibition of AChE in vitro	1.05 µM	[51]
				sporothrin B (**81**), sporothrin C (**82**), diaporthin (**83**)	HepG2	20, 23, and 23 µg/mL	
**31**	*Eurotium rubrum*	Semi-mangrove plant *Hibiscus tiliaceus*	Hainan Island, China	12-demethyl-12-oxo-eurotechinulin B (**84**), 9-dehydroxyeurotinone (**85**), variecolorin G (**86**), alkaloid E-7 (**87**), and emodin (**88**)	HepG2, MCF-7, SW1990, HepG2, NCI-H460, SMMC7721, HeLa, and Du145	In the range of 15–30 µg/mL	[52]
**32**	*Bionectria ochroleuca*	Inner leaf tissues of the plant *Sonneratia caseolaris*	Hainan island, China	Pullularins E (**89**), F (**90**), pullularins A (**91**), and C (**92**)	L5178Y	EC_50_ values In the range of 0.1 and 6.7 µg/mL	[53]
				verticillin D (**93**)	L5178Y	<0.1 µg/mL	
**33**	*Aspergillus* sp. HN15-5D	Leaves, *Acanthus ilicifolius*	Hainan Island, China	Aspergisocoumrins A–B (**94**–**95**)	MDA-MB-435	5.08 and 4.98 µM	[54]
**34**	*Aspergillus niger* MA-132	*Avicennia marina*	Hainan, China	Nigerasterol A (**96**) Nigerasterol B (**97**)	HL60	0.30 µM, 1.50 µM	[55]
				Nigerasterol A (**96**) Nigerasterol B (**97**)	A549	1.82 and 5.41 µM	
**35**	*Aspergillus nidulans* MA-143	Leaves, *Rhizophora stylosa*		Aniquinazolines A–D (**98**–**101**)	Brine shrimp	LD_50_ 1.27, 2.11, 4.95 and 3.42 µM, (Colchicine LD_50_ 88.4 µM	[56]
**36**	*Aspergillus terreus* (No. GX7-3B)	Branch of *Bruguiera gymnoihiza* (Linn.)	South China Sea	3β,5α-dihydroxy-(22*E*,24*R*)-ergosta-7,22-dien-6-one (**102**), Beauvericin (**104**)	MCF-7, A549, HeLa and KB	4.98 and 2.02, 1.95 and 0.82, 0.68 and 1.14, 1.50 and 1.10 µM	[57]
				3β,5α,14α-trihydroxy-(22*E*,24*R*)-ergosta-7, 22-dien-6-one (**103**)	MCF-7, A549, HeLa and KB	25.4, 27.1, 24.4, 19.4 µM	
**37**	*Aspergillus terreus* (No. GX7-3B)	Branch of *Bruguiera gymnoihiza* (Linn.)	South China Sea	Botryosphaerin F (**105**) and LL-Z1271β (**106**)	MCF-7 and HL-60	4.49 and 3.43 µM	[58]
					HL-60	0.6 µM	
**38**	*Penicillium* sp. J-54	Leaves, *Ceriops tagal*	Hainan province, China	Penicieudesmol B (**107**),	K-562	90.1 µM, (paclitaxel, 9.5 µM)	[59]
**39**	*Penicillium citrinum* HL-5126	*Bruguiera sexangula* var. *rhynchopetala*	South China Sea	Penibenzophenone B (**108**)	A549 Cells	15.7 µg/mL	[60]
**40**	*Penicillium* sp.	*Panax notoginseng*	Wenshan, Yunnan province, China	Brefeldin A (**109**), Brefeldin A 7-*O*-acetate (**110**)	293, HepG2, Huh7 and KB cell line	LD_50_ value from 0.024 to 0.62 µM. Both the compounds arrested HepG2 cells at the S phase	[61]
**41**	*Penicillium chrysogenum* V11	Vein of *Myoporum bontioides*	Leizhou Peninsula, China	Penochalasin K (**111**)	MDA-MB-435, SGC-7901 and A549 cells	<10 µM	[62]
**42**	*Penicillium janthinellum* HDN13-309	*Sonneratia caseolaris*	Hainan Province, China	Penicisulfuranols A–C (**112**–**114**)	HeLa and HL-60 Cells	In the range of 0.1 to 3.9 µM	[63]
**43**	*Penicillium chrysogenum* V11	Not reported	Not reported	Penochalasin I (**115**), chaetoglobosins A (**116**), and cytoglobosin C (**117**)	MDA-MB-435 and SGC-7901 cells	<10 µM	[64]
				Compounds (**116**), and (**117**)	SGC-7901 and A549 cells	<10 μM	
**44**	*Penicillium brocae* MA-231	Mangrove plant *Avicennia marina*	Hainan Island, China	Spirobrocazine C (**118**)	A2780	59 µM	[65]
				Brocazine G (**119**)	A2780 and A2780 CisR	664 nM, 661 nM (cisplatin 1.67 and 12.63 µM)	
**45**	*Penicillium brocae* MA-231	Mangrove plant *Avicennia marina*	Hainan Island, China	Brocazines A (**120**), B (**121**), E (**122**), F (**123**)	Du145, Hela, HepG2, MCF-7, NCI-H460, SGC-7901, SW1990, SW480, and U251	from 0.89 to 9.0 µM	[66]
				Compounds (**120**) and (**121**)	SW480 tumor cell line	2.0 and 1.2 µM	
				Compound (**123**)	DU145 and NCI-H460 Cells,	1.7 and 0.89 µM	
**46**	*Penicillium chermesinum* strain HLit-ROR2	*Heritiera littoralis*,	Samut Sakhon province, Thailand	TMC-264 (**124**)	T47D and MDA-MB231	1.08 and 2.81 µM (doxorubicin 1.55 and 2.24 µM)	[67]
					HepG2	3.27 µM (Etoposide, 35.66 µM)	
					MOLT-3	1.36 µM	
					T47D	1.08 µM	
				PR-toxin (**125**)	HuCCA-1, HeLa, T47D, and MDA-MB231	0.81–2.19 µM (doxorubicin, 0.26–2.24 µM)	
					HL-60 cell line	0.06 µM (doxorubicin, 1.21 µM)	
					MOLT-3 and HL-60	0.09 µM, 0.06 µM	
**47**	*Penicillium chrysogenum* HND11-24	The rhizosphere soil of the mangrove plant *Acanthus ilicifolius*	China	Penicitols A (**126**)	HeLa, BEL-7402, HEK-293, HCT-116, and A549 Cells	4.6−10.5 µM	[68]
				Penicitols B (**127**)		3.4−9.6 µM	
				Penicitols C (**128**) and Penixanacid A (**129**)		In the range of 10–40.5 µM	
**48**	*Penicillium* sp. FJ-1	*Avicennia marina*	Fujian, China	Compound (**130**)	Tca8113 and MG-63 cells	26 and 35 µM (Taxol, 46 and 10 nM)	[69]
				Compounds (**131**)	Tca8113 and WRL-68	10 and 58 µM	
				Compounds (**131**)	MG-63 cells	55 nM	
**49**	*Penicillium* sp. GD6,	*Bruguiera gymnorrhiza*	Zhanjiang, China	Meleagrin (**132**)	HL60 and A549	9.7 and 8.3 µM	[70]
**50**	*Penicillium* 303#	Sea water	Guangdong Province, China	5S, 7R, 9S, 10S, 11R, 12S, 13R, 22R, and 23R. (**133**), 7R, 9S, 10S, 11R, 12S, 13R, 22R, and 23R (**134**)	MDA-MB-435, HepG2, HCT-116, and A549	In the range of 11.9–37.82 µg/mL	[71]
				Compounds (**135**)	MDA-MB-435	7.13	
				Compound (**136**)	HepG2 and HCT-116	39.64 and 27.80 µM	
**51**	*Penicillium* sp. ZH58	Leaves, *Avicennia* sp.	Dong Sai, Hainan of the South China Sea coast	4-(methoxymethyl)-7-methoxy-6-methyl-1(3*H*)-isobenzofuranone (**137**)	KB and KB_V_200 cells	6 and 10 µg/mL	[72]
**52**	*Penicillium sumatrense* MA-92	Rhizosphere, *Lumnitzera racemose*	WenChang in Hainan Island, China	Sumalarins A–C (**138**, **139**, **140**), and dehydrocurvularin (**141**)	Du145, HeLa, Huh 7, MCF-7, NCI-H460, SGC-7901, and SW1990 Cells	In the range of 3.8 to 10 µM	[73]
**53**	*Penicillium* sp. ZH16	*Avicennia* sp.	South China Sea	5-methyl-8-(3-methylbut-2-enyl) furanocoumarin (**142**)	KB and KB_V_200	5 and 10 µg/mL	[74]
**54**	*Trichoderma* sp. 307	Stem bark, *Clerodendrum inerme*	Guangdong Province, China	(3*S*)-6-oxo-de-*O*-methyllasiodiplodin (**143**)	GH3 and MMQ Cells RPC	21.42 and 13.59 µM, 142.8 µM	[75]
	Co cultured with *Acinetobacter johnsonii* B2			(3*R*)-de-*O*-methyllasiodiplodin (**144**)		6.44 and 6.58 µM, 6.94 µM	
				(3*R*)-nordinone (**145**)		12.33 and 10.13 µM, 100.03 µM.	
**55**	*Trichoderma* sp. 307 co-culturing with *Acinetobacter johnsonii* B2	Stem bark of *Clerodendrum inerme*	Guangdong Province, China	Botryorhodine H (**146**)	MMQ GH3 Cells	3.09 and 3.64 µM	[76]
**56**	*Trichoderma* sp. Xy24	Leaves, stems and peels of *Xylocarpus granatum*	Hainan province, China	(9*R*,10*R*)-dihydro-harzianone (**147**)	HeLa and MCF-7 Cells	30.1 µM and 30.7 µM	[77]
**57**	*Nigrospora* sp. MA75	*Pongamia pinnata*	Guangxi Zhuang Autonomous Region of China	2,3-didehydro-19α-hydroxy-14-epicochlioquinone B (**148**)	MCF-7, SW1990, and SMMC7721	4, 5, and 7 µg/mL	[78]
**58**	*Fusarium* sp. (No. DZ27)	Bark of *Kandelia candel*	Dongzhai mangrove, Hainan, China	Beauvericin (**104**)	KB and KBv200 cells	5.76 and 5.34 µM	[79]
**59**	*Fusarium* sp.	Leaf of mangrove *Kandelia candel*	Dongzhai Harbor of Hainan Island, China	Apicidin (**149**)	GLC-82 cells	6.94 µM	[80]
**60**	Unidentified fungus ZZF42	South China Sea	Not reported	Apicidin (**149**)	KB and KBv200	0.78 µg/mL	[81]
**61**	*Fusarium incarnatum* (HKI0504)	*Aegiceras corniculatum*	Not reported	2-acetyl-1,2,3,4-tetrahydro-β-carboline (**150**)	HUVEC and K-562	GI_50_ 41.1 and 33.3	[82]
					HeLa cell	CC_50_ 23.8 µM	
	*Fusarium incarnatum* (HKI0504)	*Aegiceras corniculatum*	Not reported	Fusamine (**151**)	HUVEC and K-562	GI_50_ 37.3 and 37.6	[82]
					HeLa cell	CC_50_ 23.3 µM	
	*Fusarium incarnatum* (HKI0504)	*Aegiceras corniculatum*	Not reported	3-(1-aminoethylidene)-6-methyl-2*H*-pyran-2,4(3*H*)-dione (**152**)	HUVEC and K-562	GI_50_ 41.1 and 33.3	[82]
					HeLa cell	CC_50_ 23.8 µM	
**62**	*Acremonium* sp.	leaves of *Sonneratia caseolaris* c	Dong Zhai Gang Mangrove Garden, Hainan, China	Torrubiellin B (**153**)	Cisplatin sensitive Cal27, Kyse510, HCC38, A2780, MDA-MB-231	In the range of 0.3 to 1.5 µM	[83]
					Cisplatin resistant, Cal27, Kyse510, HCC38, A2780, MDA-MB-231	In the range of 0.2 to 2.6 µM	
**63**	*Acremonium strictum*	*Rhizophora apiculata*	Island of Cat Ba, Vietnam	Waol A (**154**), Pestalotiopene A (**155**) Cytosporone E (**156**)	Cisplatin-sensitive, A2780	27.1, 76.2, and 8.3 µM	[84]
					Cisplatin-Resistant A2780	12.6, 30.1, and 19.0 µM	
**64**	Endophytic fungus J3	*Ceriops tagal*	Hainan province, China	3,4-seco-sonderianol (**157**)	K562, SGC-7901, and BEL-7402 Cells	9.2, 15.7, and 25.4 µg/mL	[85]
**65**	Endophytic fungus No. ZH-3	Not reported	South China Sea	2-(3-chloro-2, 6-dihydroxy-4-methylbenzoyl)-5-hydroxy-3-methoxybenzoate (**158**)	HepG2 cell line	25 µg/mL	[86]
**66**	Endophytic fungus No. 5094	Not reported	South China Sea	Anthracene derivative (**159**)	KB and KBv200	LD_50_ values of 5.5 and 10.2 µM	[87]
**67**	Co-cultures of two mangrove endophytic fungi (strains Nos. 1924 and 3893)	Not reported		Marinamide (**160**)	HepG2, 95-D, MGC832 and HeLa Cells	7.0, 0.4, 91 nM and 0.529 µM	[88]
				Methyl marinamide (**161**)	HepG2, 95-D, MGC832 and HeLa Cells	2.52, 1.54 13, 0.110 µM	
**68**	Endophytic fungus No·Gx-3a	Not reported	South China sea	Ditryptophenaline (**162**)	KB, KBv200	8.0 and 12.0 µM	[89]
**69**	Mangrove endophytic fungus No·SK7RN3G1	Not reported	South China Sea	3,8-dihydroxy-6-methyl-9-oxo-9*H*-xanthene-1-carboxylate (**163**), Lichenxanthone (**164**),	HepG2 cell line	20 and 25 µg/mL	[90]
Compounds Produced by *Basidiomycetes*							
**70**	Basidiomycetous fungus XG8D	leaves of *Xylocarpus granatum*	Samutsakorn province, Thailand	Merulinols C and D (**165**, **166**)	KATO-3 cells	35.0 and 25.3 µM	[91]
**71**	*Pseudolagarobasidium acaciicola*,	*Bruguiera gymnorrhiza*	Samut Sakhon province, Thailand	Compound (**167**)	HuCCA-1, A549, MOLT-3, HepG2, MDA-MB231, T47D	0.28–37.46 µM	[92]
					MRC-5	IC_50_ 17.92 µM	
					HL-60 cell line	IC_50_ 0.28 µM	
				Compound (**168**)	A549, MOLT-3, HepG2, HL-60, MDA-MB231, T47D, HeLa cancer cell, MRC-5	12.09–170.08 µM	
				Compound (**169**)	HuCCA-1, A549, MOLT-3, HepG2, HL-60, MDA-MB231, T47D, HeLa cancer cell	15.20–76.97 µM	
				Compound (**170**)	HuCCA-1, A549, MOLT-3, HepG2, MDA-MB231, T47D, HeLa cancer cell	18.31–154.51 µM	
					HL-60	18.31 µM	
**72**	*Pseudolagarobasidium acaciicola*	*Bruguiera gymnorrhiza*	Not reported	Endoperoxide (**171**), Steperoxide A (**172**)	MOLT-3, HuCCA-1, A549, HepG2, HL-60, MDA-MB-231, T47D, and HeLa cancer Cells	In the range of 0.68–3.71 and 0.67–5.25 µg/mL	[93]
				Merulin B (**173**)	MOLT-3, A549, HepG2, HL-60, MDA-MB-231 and T47D Cells	In the range of 11.94–49.08 µg/mL	
				Merulin C (**174**)	HL60 cancer cells	0.08 µg/mL	
					MOLT-3, HuCCA-1, A549, HepG2, MDA-MB-231, T47D, and HeLa Cells	In the range of 0.19–3.75 µg/mL	
**73**	*Mucor irregularis* QEN-189	*Rhizophora stylosa*	Hainan Island, China	Rhizovarins A, B, E (**175**, **176**, **177**) Penitrems A, C, F (**178**, **179**, **180**) and 3β-hydroxy-4β-desoxypaxilline (**181**)	A-549	11.5, 6.3, 9.2, 8.4, 8.0, 8.2 and 4.6 µM	[94]
				Rhizovarins A, B, (**175**, **176**), Penitrems A, C, F (**178**, **179**, **180**) and 3β-hydroxy-4β-desoxypaxilline (**181**)	HL-60	9.6, 5.0, 7.0, 4.7, 3.3 and 2.6 µM	
